# An extended DNMA-based multi-criteria decision-making method and its application in the assessment of sustainable location for a lithium-ion batteries’ manufacturing plant

**DOI:** 10.1016/j.heliyon.2023.e14244

**Published:** 2023-03-07

**Authors:** Arunodaya Raj Mishra, Pratibha Rani, Abhijit Saha, Ibrahim M. Hezam, Fausto Cavallaro, Ripon K. Chakrabortty

**Affiliations:** a Department of Mathematics, Government College Raigaon, Satna, MP-485441, India; b Department of Engineering Mathematics, Koneru Lakshmaiah Education Foundation, Vaddeswaram, Andhra Pradesh-522302, India; c Department of Statistics & Operations Research, College of Sciences, King Saud University, Riyadh, Saudi Arabia; d Department of Economics, University of Molise, Via De Sanctis, 86100 Campobasso, Italy; e School of Engineering and IT, UNSW Canberra at ADFA, Australia

**Keywords:** Single-valued neutrosophic sets, Manufacturing plant location selection, Lithium-ion battery, Multi-criteria decision-making, DNMA, MEREC, SWARA

## Abstract

Lithium-ion battery (LiB), a leading residual energy resource for electric vehicles (EVs), involves a market presenting exponential growth with increasing global impetus towards electric mobility. To promote the sustainability perspective of the EVs industry, this paper introduces a hybridized decision support system to select the suitable location for a LiB manufacturing plant. In this study, single-valued neutrosophic sets (SVNSs) are considered to diminish the vagueness in decision-making opinions and evade flawed plant location assessments. This study divided into four phases. First, to combine the single-valued neutrosophic information, some Archimedean-Dombi operators are developed with their outstanding characteristics. Second, an innovative utilization of the Method based on the Removal Effects of Criteria (MEREC) and Stepwise Weight Assessment Ratio Analysis (SWARA) is discussed to obtain objective, subjective and integrated weights of criteria assessment with the least subjectivity and biasedness. Third, the Double Normalization-based Multi-Aggregation (DNMA) method is developed to prioritize the location options. Fourth, an illustrative study offers decision-making strategies for choosing a suitable location for a LiB manufacturing plant in a real-world setting. Our outcomes specify that Bangalore (*L*_2_), with an overall utility degree (0.7579), is the best plant location for LiB manufacturing. The consistency and robustness of the presented methodology are discussed with the comparative study and sensitivity investigation. This is the first study in the current literature that has proposed an integrated methodology on SVNSs to select the best LiB manufacturing plant location by estimating both the objective and subjective weights of criteria and by considering ambiguous, inconsistent, and inexact manufacturing-based information.

## 1 Introduction

*Electric Vehicles (EVs)* are a promising technology for achieving sustainable transportation in the future due to several factors, including price reduction as well as climate and environmental awareness [1,2]. The EVs will consider about 20% of the worldwide road transport fleet by 2040 [3]. At present, *Lithium-ion batteries (LIBs)* are the most appropriate *energy storage technology (EST)* for powering the EVs owing to their outstanding characteristics, including high energy efficiency and high power density [4]. The rigid demands on transportation fuels and their devastation to the atmosphere make the need for a super battery, motivating the recent extreme increase in the LIBs market (Alfaro-Algaba et al., 2020) [5]. On the other hand, when a LiB capacity denigrates to 70%–80% of its original capability, it can no longer activate an automobile and should be interchanged [6]. Assessment of utilized LiBs has become a key concern in the transportation sector and academia [7]. *Remanufacturing, repurposing and recycling (3R)* are three dominant management systems for utilized LiBs. Remanufacturing is the furthermost eco-friendly process as it conserves the individuality of LiBs by exchanging imperfect or out-of-date cells/components [8], which is a prime approach for the sustainability of the EVs sector, refining commercial competitiveness and decreasing environmental weights [9]. Also, it positively influences the diverse aspects of sustainable development [3].

Nearly 25% of the latest LiB manufacturing could be exchanged for remanufacturing by 2030 [10]. LiB manufacturing is an industrialized value-worth procedure of altering a utilized battery, in any case, its actual assessment by substituting flawed or out-of-date cells/components. It comprises the evaluation, surface cleaning, renovating, and analyzing the LiBs in a quasi-new form to fulfill the entire benchmarks enforced by actual equipment producers. It is observed that LiB manufacturing is an important influence to achieve the *circular economy (CE)* standard. However, several issues/problems still occur that obstruct large-scale remanufacturing procedures in industrial practices [2,11]. *Consequently, the current work raises some key research concerns: (i) which assessment sustainability indicators impact the considered decisions on locating LiB manufacturing/remanufacturing plants? And (ii) how to obtain a suitable LiB manufacturing plant location with uncertainty?*

With the presence of several tangible and intangible criteria, selection of the desirable location for LiB manufacturing plant can be considered as an uncertain and complex *multi-criteria decision-making (MCDM)* problem. Zolfani et al. [12] developed a hybrid model based on Bayesian best-worst method and measurement of alternatives and rankings according to compromise solution approach for selecting the most suitable location for lithium battery industry. For evaluating the locations for LiB manufacturing plant, Asaba et al. [13] extended the two-dimensional cost–knowledge model given by Duffner et al. [14] by exploring the role of clean energy, costs, and knowledge on location decisions in Europe. In general, real-world problems are defined under uncertain, imprecise or vague conditions [15,16]. In order to handle the uncertainty and vagueness of real-life decision-making problems, the concept of *fuzzy set (FS)* has widely been used in the literature [17]. Later, several generalization of FS have been introduced with wider applications [18,19]. As an extended version of FS, the notion of *intuitionistic fuzzy set (IFS)* [20] has been introduced, which describes the uncertain and vague characteristics of the things more precisely. However, the theory of IFS is not suitable for the indeterminate and inconsistent information embedded in practical situations. To handle this information, the concept of *Neutrosophic set (NS)* [21] has found to be more valuable tool. It is characterized by the *truth membership function (TMF)*, *indeterminacy membership function (IMF)* and *falsity-membership function (FMF)*, where all the functions are totally independent and lie in $]0^{-},1^{+}[\text{.}$ With the use of NS theory, Deveci et al. [2] designed a decision support system for identifying and choosing the most desirable location for automotive LiB remanufacturing plant.

As the generalization of NS, *single-valued neutrosophic set (SVNS)* [22] is a more functional tool to handle the incomplete, indeterminate and inconsistent information that usually exists in real scientific and engineering applications. Inspired by the idea of SVNS theory, we develop an integrated MCDM methodology under single-valued neutrosophic environment. This method uses to find the suitable location selection for a LiB manufacturing plant and encourages the sustainable perspectives of the EVs sector. The proposed methodology combines the *method based on the removal effects of criteria (MEREC)*, *stepwise weight assessment ratio analysis (SWARA)* and *double normalization-based multi-aggregation (DNMA)* approaches from single-valued neutrosophic perspective. This paper proposes LiB manufacturing plant selection by edifying assessment parameters/factors/criteria for locating the manufacturing plants and offering a comprehensive MCDM methodology for professionals.

The key challenges are identified from extant literature, given as

a) Main strategic assessment parameters that impact the selection of LiB manufacturing plant locations are still missing in the literature [2,9];
b) While several MCDM models for manufacturing have been discussed by several investigators, however, very few models used in the manufacturing discipline considering the uncertain, vagueness, imprecise and inexact information;
c) The DNMA approach [23] is a prominent and significant tool, which uses the subordinate degree and ranks the alternatives by proposed aggregation operators. However, the DNMA approach has not been developed previously on SVNSs setting;
d) The key concern in locating a LiB manufacturing plant is to estimate the weight values of criteria/parameters. The MEREC is an objective weighting tool pioneered by Keshavarz-Ghorabaee et al. [24], and SWARA is a subjective weighting tool proposed by Kersuliene et al. [25], which has attracted notable concentration from scholars in diverse areas due to its consistency and precision. To date, no earlier study has used the combination of MEREC-SWARA in the manufacturing and decision-making discipline.

To overcome the limitations of existing studies, this study presents the following key contributions.

(i) Identifying the main criteria for assessing manufacturing plant location for LiBs with sustainability perspectives.
(ii) Implementing an MCDM problem by simultaneously considering uncertain, indeterminate, and inconsistent information. Consequently, an integrated SVN-MEREC-SWARA-DNMA approach is presented.
(iii) An integrated weighting approach for criteria weights is proposed. In this regard, objective weight is derived by the MEREC, and the subjective weight is determined through the SWARA method. This combination conquers the shortcomings that arise with either an objective-weighting or a subjective-weighting procedure.
(iv) To offer appropriate generalization and flexibility during the fusion process, this paper further introduces Archimedean-Dombi operational laws and related weighted aggregation operators by combining Archimedean and Dombi operations' with *single-valued neutrosophic numbers (SVNNs).*
(v) The presented SVN-MEREC-SWARA-DNMA model is applied on an illustrative assessment of manufacturing plant location for LiBs with sustainability perspectives on SVNSs. Here, we discuss the *economic, environmental, social and technical (EEST)* dimensions of sustainability to select the manufacturing plant location for LiBs.

The remaining sections are scheduled as: Section 2 confers the previous studies related to this study. Section 3 shows the fundamentals of SVNSs and then presents Archimedean-Dombi weighted aggregation operators for combining the SVNNs. Section 4 introduces an integrated SVN-MEREC-SWARA-DNMA model to treat the decision-making problems under SVNS context. Section 5 shows a manufacturing plant location assessment case study for LiBs from SVNS perspective. Further, this section approves the strength of the presented approach with the comparison and sensitivity study. Section 6 presents the concluding results and recommends future research perspective.

## 2 Literature review

This section discusses comprehensive works related to SVNSs, weighting methods, DNMA, and MCDM approaches for manufacturing sectors.

### 2.1 Studies related to SVNSs

Several theories and concepts have been introduced on FS theory, but these philosophies cannot treat the indecision of *decision experts (DEs)* as it is solely described by a membership function. To conquer the insufficiency of FS, the idea of IFS has pioneered by Atanassov [20]. However, IFSs can only deal with inadequate and uncertain information. To overcome the drawbacks of IFSs and handle the uncertain, indeterminate and inconsistent information, Smarandache [21] pioneered the NS theory that comprises three factors: TMF, IMF and FMF. Afterwards, numerous researches have been developed under the concepts of NSs [2,[26], [27], [28]].

Generally, applying the NSs in realistic settings is challenging since the TMF, IMF, and FMF lies in ]0^−^, 1^+^[. To conquer this concern, the idea of “single-valued neutrosophic set (SVNS)” and its properties have been intended by Wang et al. [22]. Chaw et al. [29] proposed an innovative *single-valued neutrosophic (SVN)* relation based MCDM methodology for choosing the significant aspects that have an effect on oil prices. Han et al. [30] investigated a SVN-based optimization approach for dealing with system uncertainty of the zinc electrowinning process. Garg and Nancy [31] put forward a group MCDM approach by considering the distance measures of SVNSs. In addition, they presented a novel clustering model to classify the objects. Al Akara et al. [32] presented the concept of SVNS in ordered semigroups wherein various ideas concerning SVN ideals and bi-ideals have been considered. In a study, Stanujkic et al. [33] put forward an innovative *single-valued neutrosophic evaluation based on distance from average solution (SVN-EDAS)* method for handling the complex MCDM applications. Mishra et al. [34] originated a hybrid SVN-based MCDM model to evaluate low-carbon tourism strategies because of different conflicting indicators and economic, environmental and social facets of sustainability. An integrated single-valued neutrosophic MCDM method based on decision-making and trial evaluation laboratory-analytical network process, geographic information system and SVN-EDAS has proposed to assess the two-stage optimal site selection for waste-to-energy plant [35]. Recently, Farid and Riaz [36] proposed new Einstein interactive aggregation operators and their application in MCDM problems under SVN environment. However, there is no study regarding the selection of LiB manufacturing plant location under indeterminate, inconsistent and uncertain environment.

### 2.2 Weighting methods

In the MCDM approaches, the criteria weights are vital aspects for the DEs [[37], [38]]. The objective weights of criteria are determined from the decision-matrix and are derived according to the information presented by the DEs [34]. Some popular objective weight determination approaches are “entropy-based method” [39], “criteria importance through inter-criteria correlation” [38], “criterion impact loss” [40], “integrated determination of objective criteria weights” [40]. Just a while ago, Keshavarz-Ghorabaee et al. [24] put forward the idea of a new objective weighting model, named as MEREC. They studied the stability of MEREC in comparison with existing objective weighting models. Since its appearance, Ghosh and Bhattacharya [41] combined the MEREC and grey-based combined compromise solution with application in tourism sector. In a study, Keleş [42] presented an improved MEREC model based on geometric and harmonic mean as multiplicative functions. Ulutaş et al. [43] incorporated the MEREC and simplified simple weight product method for evaluating and prioritizing the pallet trucks. Ul Haq et al. [44] studied an integrated MEREC-based ranking model for evaluating and selecting the materials from sustainable viewpoints.

While the subjective weights of criteria are estimated according to the DEs' opinions about the relative importance of criteria [45]. Kersuliene et al. [25] proposed the SWARA tool to estimate the subjective weights, which considers the DEs’ opinions related to the significance ratio of criteria. Numerous researchers have applied the SWARA tool in several areas. For example, Rani and Mishra [46] integrated the SWARA-based ranking method for handling the MCDM problems on SVNSs settings. Mohammadian et al. [47] merged the SWARA and additive ratio assessment methods with interval-valued triangular fuzzy numbers to determine and prioritize IoT application areas in the agriculture industry. Yücenur and Şenol [48] proposed a two-step framework by integrating SWARA and fuzzy ranking model for assessing lean construction techniques. Kumar et al. [49] integrated the SWARA and the combined compromise solution with its application in automobile sector. Recently, various studies have used the SWARA tool in diverse settings [[50], [51]]. For the first time, the MEREC and the SWARA models are firstly integrated with the DNMA method on SVNSs setting.

For DE's weighting model, the RS model [52] has developed to determine the significance values of DEs. Until now, no one has used the RS weighting model to compute the DE's weight on SVNSs setting to the manufacturing plant location selection for LiB. This procedure will be appropriate to assist us in assessing the considered alternatives and elucidate the significance of the DE's roles during the process of MCDM. In this line, the DE's assessment of each option with considered criteria is very significant while choosing the suitable preference. This paper applies the RS-weighting procedure to obtain the DE's weights for solving the MCDM problems.

### 2.3 Studies related to the DNMA method

The DNMA [23] is one of the novel and effective utility theory-based method, which incorporates the two different normalization processes and three aggregation techniques. For the first time, the merits of DNMA method has been emphasized in comparison with other utility-theory-based MCDM methods [53]. Further, Liao et al. [54] suggested a hesitant fuzzy information-based DNMA technique for solving the lung cancer screening problem. Nie et al. [55] designed an innovative decision support system with the integration of cardinal consensus process, DNMA method and hesitant linguistic term set. Moreover, their application has presented in the assessment of geographical sites for shopping mall with hesitant linguistic information. Wang and Rani [56] combined the DNMA method and intuitionistic fuzzy information for evaluating and prioritizing the sustainability risks in supply chain management. Rahimi et al. [57] extended the classical DNMA method under interval-valued Pythagorean fuzzy context and applied to assess the digital transformation challenges in sustainable financial service systems. In the recent past, Hezam et al. [58] assessed the barriers of digital sustainable transportation for disabled persons using Fermatean fuzzy DNMA method.

### 2.4 MCDM approaches for manufacturing

Remanufacturing fascinated huge attention in academia and among researchers in recent times. Consequently, numerous MCDM tools have been developed to treat different manufacturing problems. Govindan et al. [59] assessed the decisive barriers to India's automotive components remanufacturing (ACR) sector. Bhatia and Srivastava [60] discussed the outer barriers in electronic waste remanufacturing. Ansari et al. [61] recognized the crucial success aspects for remanufacturing adoption in manufacturing sectors and ranked the performance results. In recent times, Ansari et al. [62] developed a systematic tool to diminish supply chain based risks in systems considered in remanufacturing practices. Deveci et al. [2] introduced a three-way MCDM tool for site assessment of a LiB remanufacturing plant on neutrosophic sets. They used the “best-worst method (BWM)” and the “combinative distance-based assessment (CODAS)” method on type-2 neutrosophic numbers to prioritize LiB manufacturing plant location selection.

### 2.5 Research gaps

Based on the earlier discussion, the research gaps are given as: *a*) *There have not been any studies to select the best LiB manufacturing plant location using the integrated methodology of the SVNSs-based decision-making model*; *b*) *no study has estimated the objective and subjective weights of criteria for locating LiB manufacturing plants*; *c*) *the available crisp, probabilistic and/or fuzzy decision-making tools for manufacturing could produce erroneous plant site selection decisions as they are not capable of treating the high levels of ambiguous, inconsistent, and imprecise manufacturing-based information*; *d*) *no study has used the SVN-MEREC-SWARA-DNMA in the manufacturing discipline*.

### 2.6 Problem definition

Battery manufacturing signifies a vital economic prospect for India. Aspiring goals, intensive policies, and a collective method could assist India to fulfill the EV goals while evading import dependence on battery packets and cells. The LiB is a key part of an EV, which delivers the essential energy storage because of the advantage of “high energy density”, “high output voltage”, “low self-discharge rate”, and “long cycling life”. Presently, the LiB manufacturing sector in India is in a nascent phase. The country grasps the potential to arise as a vital producer of LiBs over the coming years. India can continue to grow the LiB manufacturing sectors in three different phases [63]: the first phase (2017–2020), the second phase (2021–2025), and the third phase (2026–2030). The primary attention is to construct an ambient manufacturing atmosphere in the initial phase. In the second phase, India is projected to capture around 25%–40% of the total economic opportunity for LiB manufacturing. The manufacturing sector is expected to reinforce its supply chain network (SCN) and create ample investments in research and development (R&D) by 2025. In this duration, India is projected to be participated in the manufacturing of battery packs, with limited manufacture of battery cells. In the third phase, producers are estimated to be involved in the end-to-end manufacture of LiBs.

**Fig. 1:**
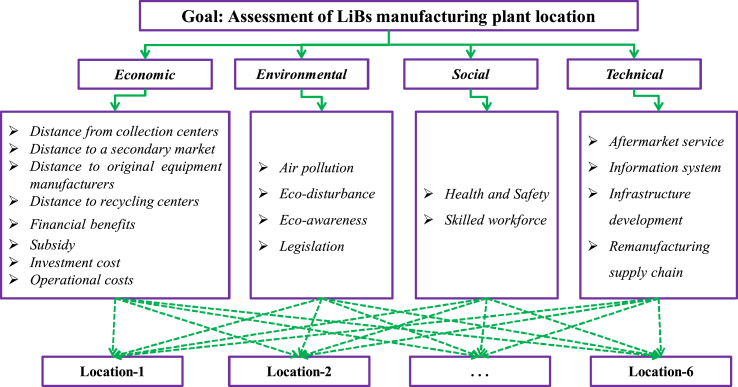
Hierarchical structure for the LiBs' manufacturing plant location.

Consequently, the dependence on imports is expected to be diminished considerably at this phase. This phase is expected to be of supreme significance for the nation to begin its individuality in the electric mobility zone by appealing to the construction of both EV and EV batteries on the domestic stage. With the preliminary utilization of EVs in India, there is a requirement to establish a LiB manufacturing plant. Four DEs from Indian organizations who are handling the energy storages and EVs are asked to suggest appropriate locations for the locating LiB manufacturing plant and create realistic and logical assessments of these locations. The considered parameters are presented in Table 1 and Fig. 1. A committee of 4 experts is considered to make a suitable decision. Six prospective locations are recognized as follows: Mumbai (*F*_1_), Bangalore (*F*_2_), Gujarat (*F*_3_), Haryana (*F*_4_), Andhra Pradesh (*F*_5_), and New Delhi (*F*_6_). The key idea of the study is to recognize the indicators/criteria for locating LiB manufacturing plants. Eighteen criteria are considered and described in Table 1 and Fig. 1. According to their nature, they are gathered into four dimensions: 8 economic, 4 environmental, 2 social and 4 technical indicators for locating LiBs manufacturing plants.

**Table 1:** Assessment criteria for locating the LiB manufacturing plants.

Dimensions	Criteria	Type	Sources
Economic	Distance from collection centers (*D*_1_)	Min	[[64], [65], [66], [67], [68]]
	Distance to a secondary market (*D*_2_)	Min	[64,65,[67], [68], [69], [70], [71]]
	Distance to original equipment manufacturers (*D*_3_)	Min	[68,[70], [71], [72], [73]]
	Distance to recycling centers (*D*_4_)	Min	[2,9,64,68,71,74]
	Financial benefit (*D*_5_)	Max	[9,61,64,[68], [69], [70],^73^]
	Subsidy (*D*_6_)	Max	[2,60,65,70]
	Investment cost (*D*_7_)	Min	[2,64,67,70,71]
	Operational costs (*D*_8_)	Min	[2,69,71,72,74]
Environmental	Air pollution (*D*_9_)	Min	[2,67,70,72]
	Eco-disturbance (*D*_10_)	Min	[2,67,68,75]
	Eco-awareness (*D*_11_)	Max	[2,60,69,70]
	Legislation (*D*_12_)	Max	[2,9,59,62,68]
Social	Health & safety (*D*_13_)	Max	[1,60,62,68]
	Skilled workforce (*D*_14_)	Max	[2,9,62,76]
Technical	Aftermarket service (*D*_15_)	Max	[2,66,76]
	Information system (*D*_16_)	Max	[2,68,70,77]
	Infrastructure development (*D*_17_)	Max	[2,61,78]
	Remanufacturing supply chain (*D*_18_)	Max	[2,59,62,66,67]

## 3 Proposed aggregation operators (AOs)

In the current portion, we firstly discuss the fundamental ideas related to this study. Further, we propose single-valued neutrosophic Archimedean-Dombi aggregation operators and their enviable characteristics.

### 3.1 Preliminaries

A NS *M* on Ω is presented by the functions *TMF* $\left(TM_{M}\left(x\right)\right)\text{,}$ *IMF* $\left(IM_{M}\left(x\right)\right)\text{,}$ *FMF* $\left(FM_{M}\left(x\right)\right)\text{,}$ satisfying $M=\left\{\left(TM_{M}\left(x\right),IM_{M}\left(x\right),FM_{M}\left(x\right)\right)|x\in \Omega \right\}\text{.}$ Also, these functions are “real standard or nonstandard subsets” of $w_{1}\text{,}$ and $0^{-}\leq \sup\left(TM_{M}\left(x\right)\right)+\sup\left(IM_{M}\left(x\right)\right)+\sup\left(FM_{M}\left(x\right)\right)\leq 3^{+}\text{.}$ Further, Wang et al. [22] proposed the idea of SVNS and its characteristics, which is an extended version of NS. All supporting definitions and their explanations are highlighted in Appendix A.

### 3.2 Single-valued neutrosophic Archimedean-Dombi operators

Inspired by *Archimedean-Dombi operations (ADOs)* for hesitant fuzzy elements [79], we present ADOs on SVNNs and further develop “single-valued neutrosophic Archimedean-Dombi operations (SVN-ADOs)” in Supplementary file S.1. Similarly, *SVN Archimedean-Dombi weighted averaging (SVN-ADWA*” and *SVN Archimedean-Dombi weighted geometric (SVN-ADWG)* operators and their properties are highlighted in Supplementary file S.2. Readers are requested to read those sections to understand all proposed operators.

## 4 Introduced SVN-MEREC-SWARA-DNMA approach

**Fig. 2:**
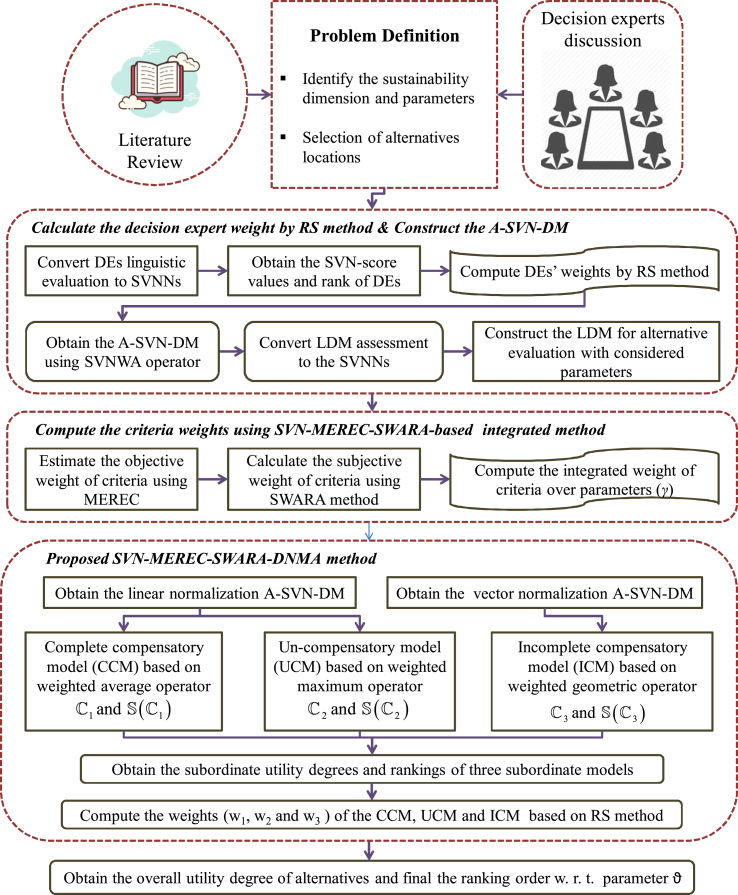
Graphical representation of SVN-MEREC-SWARA-DNMA approach.

This section introduces a new extension of the DNMA method with SVN-ADW operators, the SVN-MEREC and the SVN-SWARA models, and named as SVN-MEREC-SWARA-DNMA. The computational procedure of the present SVN-MEREC-SWARA-DNMA framework is specified as (see Fig. 2).

**Step 1:** Construct the linguistic assessment matrix (LAM).

Consider the sets of options/alternatives $\left\{F_{1},F_{2},...,F_{m}\right\}$ and attributes/criteria $\left\{D_{1},D_{2},...,D_{n}\right\}\text{.}$ A team of DEs $\left\{e_{1},e_{2},...,e_{l}\right\}$ provides the performance value of each option *F*_*i*_ over a criterion *D*_*j*_ in the form of *linguistic values (LVs)*. Let $\Omega ={\left[\chi_{ij}^{\left(k\right)}\right]}_{m\times n};\;i=1,2,...,m,j=1,2,...,n$ be the LAM, in which $\chi_{ij}^{\left(k\right)}$ represents the linguistic performance value of *F*_i_ concerning a criteria *D*_*j*_ given by *k*^th^ DE.

**Step 2:** Derive the DEs' weights using RS method.

Here, the RS weighting tool uses to determine the DE's weight, which is given as

(1) $$\varpi_{k}=\frac{l-r_{k}+1}{\sum_{k=1}^{l}\left(l-r_{k}+1\right)}\text{,}$$

wherein $\varpi_{k}\geq 0$ and $\sum_{k=1}^{l}\varpi_{k}=1$ represents the weights for each expert, *r*_*k*_ denotes the rank of *k*^th^ expert.

**Step 3:** Form the “aggregated SVN-decision-matrix (A-SVN-DM)”. To aggregate the individuals’ opinions, the SVN-ADWA or SVN-ADWG operator is employed on SVN-DM and then we construct an A-SVN-DM $N={\left({ο}_{ij}\right)}_{m\times n}\text{,}$ where

(2) $${ο}_{ij}=\left(tm_{ij},im_{ij},fm_{ij}\right)=SVNADWA_{\varpi }\left({ο}_{ij}^{\left(1\right)},{ο}_{ij}^{\left(2\right)},...,{ο}_{ij}^{\left(l\right)}\right)\text{.}$$

or

(3) $${ο}_{ij}=\left(tm_{ij},im_{ij},fm_{ij}\right)=SVNADWG_{\varpi }\left({ο}_{ij}^{\left(1\right)},{ο}_{ij}^{\left(2\right)},...,{ο}_{ij}^{\left(l\right)}\right)\text{.}$$

**Step 4:** The proposed SVN-MEREC-SWARA method for criteria weights calculation.

We integrate the objective and subjective weight-determining procedures to derive the criteria weights within the context of SVNS.

**Case I:** SVN-MEREC for objective weights.

The SVN-MEREC technique consists of given procedures.

**Step 4a:** First, we normalize the aggregated matrix into normalized form $\bar{N}={\left(\varsigma_{ij}\right)}_{m\times n}\text{,}$ where

(4) $$\varsigma_{ij}=\left(tm_{ij},im_{ij},fm_{ij}\right)=\left\{ \begin{array}{l} {ο}_{ij}=\left(tm_{ij},im_{ij},fm_{ij}\right),\;j\in D_{b}\\ {\left({ο}_{ij}\right)}^{c}=\left(fm_{ij},1-im_{ij},tm_{ij}\right),\;j\in D_{n} \end{array}\right.\text{.}$$

**Step 4b:** Create a score matrix $\Phi ={\left(\sigma_{ij}\right)}_{m\times n}$ of each single-valued neutrosophic number $\varsigma_{ij}\text{,}$ where

(5) $$\sigma_{ij}=\left(\frac{2+tm_{ij}-im_{ij}-fm_{ij}}{3}\right)\text{.}$$

Step 4c Determine the overall performance value of each option using Eq. (6).

(6) $$\vartheta_{i}=\ln\left(1+\left(\frac{1}{n}\;\sum_{j}\left|\ln\left(\sigma_{ij}\right)\right|\right)\right)\text{.}$$

**Step 4d:** The overall performance $\vartheta_{i}^{'}$ of *i*^th^ alternative by eliminating *j*^th^ attribute is determined as

(7) $$\vartheta_{i}^{\prime }=\ln\left(1+\left(\frac{1}{n}\;\sum_{k,k\neq j}\left|\ln\left(\sigma_{ik}\right)\right|\right)\right)\text{.}$$

Step 4e Assess the sum of absolute deviation by using Eq. (8).

(8) $$Abd_{j}=\sum_{i}\left|\vartheta_{ij}^{\prime }-\vartheta_{i}\right|\text{.}$$

**Step 4f:** Obtain the objective weight of the *j*^th^ criterion using

(9) $$w_{j}^{o}=\frac{Abd_{j}}{\sum_{j=1}^{n}Abd_{j}},\;\forall \;j\text{.}$$

**Case II:** SVN-SWARA for subjective weights.

To find the subjective weights by SVN-SWARA model, we firstly determine the score values of aggregated performance values of criteria, which are presented by the DEs. Then, we rank the criteria from high to low score values.

**Step 4g:** From the second criterion, comparative importance of average values (*c*_*j*_) should be done as follows: the relative importance of criterion ‘*j*’ in relation to the previous ‘*(j-1)*’ criterion.
**Step 4d:** Compute the coefficient using Eq. (10).

(10) $$k_{j}=\left\{ \begin{array}{l} 1,\;j=1,\\ c_{j}+1,\;j\;>\;1. \end{array}\right.$$

**Step 4e:** Compute the initial weight of each criterion.

(11) $$g_{j}=\left\{ \begin{array}{l} 1,\;j=1,\\ \frac{g_{j-1}}{k_{j}},\;j\;>\;1. \end{array}\right.$$

**Step 4f:** Estimate the normalized weight of each criterion.

(12) $$w_{j}^{s}=\frac{g_{j}}{\sum_{j=1}^{n}g_{j}},\forall \;\;j.$$

***Case III:****U*sing Case I and Case II, we estimate the final weights of criteria by Eq. (13).

(13) $$w_{j}=\gamma \;w_{j}^{o}+\left(1-\gamma \right)\;w_{j}^{s},\;\forall \;j\text{,}$$

where γ denotes the “decision precision coefficient” and γ∈[0,1].

**Step 5:** Assess the “normalized A-SVN-DM (NA-SVN-DM)”

A linear normalization process is given as

(14) $${\mathbb{N}}^{\left(1\right)}={\left(\eta_{ij}^{\left(1\right)}\right)}_{m\times n},\;where\;\eta_{ij}^{\left(1\right)}=\left(\bar{t}m_{ij}^{\left(1\right)},\bar{i}m_{ij}^{\left(1\right)},\bar{f}m_{ij}^{\left(1\right)}\right)=\left\{ \begin{array}{l} \frac{o_{ij}}{\max_{i}\;S\left(o_{ij}\right)},\;j\in D_{b}\\ 1-\frac{o_{ij}}{\max_{i}\;S\left(o_{ij}\right)},\;j\in D_{n} \end{array}\right.$$

where S(.) denotes the score value of a SVNN.

The vector normalization is utilized to normalize the A-SVN-DM into $N^{\left(2\right)}={\left(\eta_{ij}^{\left(2\right)}\right)}_{m\times n}\text{,}$ where

(15) $$\eta_{ij}^{\left(2\right)}=\left\{ \begin{array}{l} \left(\bar{t}m_{ij}^{\left(2\right)},\bar{i}m_{ij}^{\left(2\right)},\bar{f}m_{ij}^{\left(2\right)}\right),\;j\in D_{b},\\ \left(\bar{f}m_{ij}^{\left(2\right)},1-\bar{i}m_{ij}^{\left(2\right)},\bar{t}m_{ij}^{\left(2\right)}\right),\;j\in D_{n}, \end{array}\right.$$

such that

(16) t‾mij(2)=tmij(∑i=1m{(tmij)2})12,i‾mij(2)=imij(∑i=1m{(imij)2})12andf‾mij(2)=fmij(∑i=1m{(fmij)2})12,∀j.

**Step 6:** Using the subordinate aggregation models.
**Step 6.1:** Process of *complete compensatory method (CCM).*

The CCM can be presented using the SVN-ADWA operator

(17) $$C_{1}=\left(\hat{t}m_{ij}^{\left(1\right)},\hat{i}m_{ij}^{\left(1\right)},\hat{f}m_{ij}^{\left(1\right)}\right)=SVNADWA_{w_{j}}\left(\eta_{i1}^{\left(1\right)},\eta_{i2}^{\left(1\right)},...,\eta_{in}^{\left(1\right)}\right)$$

wherein $w_{j}$ and $\eta_{ij}^{\left(1\right)}$ show the criterion weight and the linear normalization value, respectively. In accordance with the decreasing values of $C_{1}\text{,}$ we get the ranking result $\rho_{1},\;i=1,2,...,m$.

**Step 6.2:** Process of un-compensatory method (UCM).

The UCM can be computed by

(18) $$C_{2}=\left(\hat{t}m_{ij}^{\left(2\right)},\hat{i}m_{ij}^{\left(2\right)},\hat{f}m_{ij}^{\left(2\right)}\right)=\max_{j}\;w_{j}{\left(\eta_{ij}^{\left(1\right)}\right)}^{c}\text{.}$$

The options can be ordered by setting up the values of $C_{2}$ in decreasing order and we obtain the ranking result $\rho_{2},\;i=1,2,...,m$.

**Step 6.3:** Process of incomplete compensatory method (ICM).

The ICM can be derived using SVNADWG operator

(19) $$C_{3}=\left(\hat{t}m_{ij}^{\left(3\right)},\hat{i}m_{ij}^{\left(3\right)},\hat{f}m_{ij}^{\left(3\right)}\right)=SVNADWG_{w_{j}}\left(\eta_{i1}^{\left(2\right)},\eta_{i2}^{\left(2\right)},...,\eta_{in}^{\left(2\right)}\right)\text{,}$$

wherein $w_{j}$ denote the criterion weight and $\eta_{ij}^{\left(2\right)}$ denote the vector normalized value. The options can be ordered according to the decreasing values of $C_{3}$ and we attain the ranking result $\rho_{3},\;i=1,2,...,m$.

**Step 7:** Integration of *subordinate utility degrees (SUDs)* and priority orders.

Each alternative *F*_*i*_ has the SUD $C_{\tau }:\tau =1,2,3$ and the preference order $\rho_{\tau }:\tau =1,2,3$ over each attribute parameters. Clearly, we form the SUD decision matrix $ƛ\left(C\right)={\left[C_{i\tau }\right]}_{m\times 3}$ and the ranking decision matrix $ƛ\left(\rho \right)={\left[\rho_{i\tau }\right]}_{m\times 3}$.

The normalized form is presented as

$${\mathbb{C}}_{\tau }^{\left(N\right)}=\left(\hat{t}m_{ij}^{\left(N\right)},\hat{i}m_{ij}^{\left(N\right)},\hat{f}m_{ij}^{\left(N\right)}\right),\;\tau =1,2,3\text{,}$$

where

tmˆij(N)=tmˆij(τ)(∑τ=13{(tmˆij(τ))2})12,imˆij(N)=imˆij(τ)(∑τ=13{(imˆij(τ))2})12

and

(20) fmˆijN=fmˆijτ∑τ=13fmˆijτ212,τ=1,2,3,i=1,2,...,m,j=1,2,...,n.

**Step 8:** Obtain the overall utility degree (OUD).

The OUD of each option is presented by

(21) $$U_{i}=[\frac{1}{2}\;(w_{1}.\;\sqrt{\vartheta \;\left(\frac{C_{1}^{\left(N\right)}}{\max_{i}\;C_{1}^{\left(N\right)}}\right)+\left(1-\vartheta \right)\;{\left(\frac{m-\rho_{1}+1}{m}\right)}^{2}}-w_{2}\;.\;\sqrt{\vartheta \;\left(\frac{C_{2}^{\left(N\right)}}{\max_{i}\;C_{2}^{\left(N\right)}}\right)+\left(1-\vartheta \right)\;{\left(\frac{\rho_{2}}{m}\right)}^{2}}+w_{3}\;.\;\sqrt{\vartheta \;\left(\frac{C_{3}^{\left(N\right)}}{\max_{i}\;C_{3}^{\left(N\right)}}\right)+\left(1-\vartheta \right)\;{\left(\frac{m-\rho_{3}+1}{m}\right)}^{2}})+1]\text{,}$$

wherein *w*_1_, *w*_2_ and *w*_3_ are the significance values of CCM, UCM, and ICM, correspondingly, with $w_{1}+w_{2}+w_{3}=1\text{.}$ Here, the weights *w*_1_, *w*_2_ and *w*_3_ are obtained using the holistic procedure, that means we provide equal significance to each model. The ultimate preference set $\rho =\left\{\rho \left(F_{1}\right),\rho \left(F_{2}\right),...,\rho \left(F_{m}\right)\right\}$ is attained according to decreasing values of $U_{i},\;i=1,2,...,m$.

## 5 Case study

In the present part of study, the SVN-MEREC-SWARA-DNMA methodology is used to rank the manufacturing plant locations for LiBs from sustainability perspectives. In this line, a team of four DEs {*e*_1_, *e*_2_, *e*_3_, *e*_4_} is made to implement the presented approach to treat the manufacturing plant locations selection problem for LiBs and to recognize the best locations among a set of manufacturing plant locations for LiB. The four DEs belong from diverse disciplines with 20+ years of expertise in their field. Corresponding to the DEs' views and extant articles, the given options are evaluated over 18 considered criteria, described in Table 1 and Fig. 1. Next, Table 2, Table 3 ([45,46,51]) show the LVs to measure the relative consequence of four DEs and for prioritizing the manufacturing plant locations for LiBs over diverse criteria, and then specified in the form of SVNNs. From Table 2 and Eq. (20), the DEs’ weights are obtained using the SVN-rank sum (RS) weight procedure of each DE to the manufacturing plant locations selection for LiBs and mentioned in Table 4. Table 5 defines the linguistic decision matrix for each manufacturing plant locations *F*_*i*_ for LiBs over diverse factors in LTs as (*e*_1_, *e*_2_, *e*_3_, *e*_4_).

**Table 2:** Linguistic ratings for weighting the DEs.

LTs	SVNNs
Highly skilled (HS)	(0.90, 0.10, 0.10)
Much skilled (MS)	(0.75, 0.20, 0.25
Very skilled (VS)	(0.60, 0.35, 0.40)
Skilled (S)	(0.50, 0.45, 0.50)
Less skilled (LS)	(0.25, 0.80, 0.75)
Very less skilled (VLS)	(0.10, 0.90, 0.90)

**Table 3:** Ratings of the LiBs’ manufacturing plant location in form of LTs.

LTs	SVNNs
Highly important (HI)	(1.00, 0.00, 0.00)
Very important (VI)	(0.90, 0.10, 0.10)
More important (MI)	(0.80, 0.15, 0.20)
Important (I)	(0.70, 0.25, 0.30)
Slightly important (SI)	(0.60, 0.35, 0.40)
Average (A)	(0.50, 0.50, 0.50)
Slightly unimportant (SU)	(0.40, 0.65, 0.60)
Unimportant (U)	(0.30, 0.75, 0.70)
More unimportant (MU)	(0.20, 0.85, 0.80)
Very unimportant (VU)	(0.10, 0.90, 0.90)
Highly unimportant (HU)	(0.00, 1.00, 1.00)

**Table 4:** DE's weight for LiBs' manufacturing plant location by RS method.

DEs	LTs	SVNNs	Score values	Ranking	Weights
*e*_1_	S	(0.50, 0.45, 0.50)	0.5167	4	0.100
*e*_2_	MS	(0.75, 0.25, 0.20)	0.7667	2	0.300
*e*_3_	VS	(0.60, 0.35, 0.40)	0.6333	3	0.200
*e*_4_	HS	(0.90, 0.10, 0.10)	0.9000	1	0.400

**Table 5:** The LDM of LiBs’ manufacturing plant location for each DE.

	*F*_1_	*F*_2_	*F*_3_	*F*_4_	*F*_5_	*F*_6_
*D*_1_	(A,SU,MU,MU)	(SU,U,SU,MU)	(U,SI, SU,A)	(SU,U,SI,A)	(U,A,A,SU)	(U,SI,A,SI)
*D*_2_	(SU,U,U,MU)	(SU,U,MU,MU)	(U,A,SI,A)	(MU,U,SI,A)	(MU,U,A,SU)	(SU,A,U,SI)
*D*_3_	(A,SU,U,A)	(SU,MU,U,MU)	(MU,SU,A,SI)	(SU,U,A,A)	(MU,A,A,SU)	(A,U,SU,SU)
*D*_4_	(MU,A,SI,SU)	(VU,A,SI,SU)	(MU,SU,SI,A)	(SU,U,SU,MU)	(MU,SU,A,SU)	(VU,U,A,SU)
*D*_5_	(SI,I,A,SI)	(A,MI,I,MI)	(I,SU,SI,U)	(A,MI,SI,SU)	(MI,SI,SI,I)	(A,MI,I,MI)
*D*_6_	(SI,I,MI,I)	(I,MI,MI,MI)	(MI,I,SI,A)	(I,MI,SI,MI)	(SI,MI,I,I)	(VI,I,U,A)
*D*_7_	(U,SI,U,A)	(MU,U,U,MU)	(U,SI,A,I)	(MU,A,SI,A)	(VU,A,SU,U)	(MU,A,SI,U)
*D*_8_	(U,SU,MU,SU)	(A,U,U,MU)	(MU,SI,A,U)	(MU,SU,U,A)	(U,A,U,SU)	(VU,U,I,SU)
*D*_9_	(SU,U,MU,U)	(SU,U,MU,MU)	(U,SU,I,A)	(U,MU,VU,U)	(SU,A,MU,VU)	(MU,A,I,SU)
*D*_10_	(SU,SU,U,A)	(MU,U,SU,MU)	(U,MU,A,MU)	(U,SU,SI,MU)	(MU,A,U,VU)	(A,SU,SI,U)
*D*_11_	(I,MI,SI,A)	(A,I,MI,MI)	(I,A,SU,U)	(MI,SI,A,U)	(I,A,SU,SI)	(SU,A,VI,A)
*D*_12_	(A,VI,I,SI)	(MI,I,I,MI)	(A,SU,SI,A)	(VI,SI,SU,A)	(MI,SI,I,MI)	(I,I,A,VI)
*D*_13_	(I,VI,SI,I)	(MI,I,SI,MI)	(SI,SI,SU,A)	(I,SI,MI,U)	(MI,A,SU,SI)	(VI,I,SI,U)
*D*_14_	(SI,A,I,SI)	(SI,I,I,VI)	(MI,MI,SI,A)	(A,I,MI,SU)	(MI,SU,A,SI)	(MI,A,SU,I)
*D*_15_	(I,A,MI,SI)	(A,VI,MI,I)	(MI,SI,I,A)	(MI,SU,I,A)	(I,SI,MI,SU)	(VI,SI,I,A)
*D*_16_	(MI,I,SI,A)	(SI,MI,I,SI)	(I,I,MI,A)	(MI,SU,SI,A)	(VI,I,A,SU)	(SI,A,I,MI)
*D*_17_	(SI,I,A,A)	(I,SU,SI,MI)	(U,SU,A,SI)	(MI,SI,U,SU)	(SU,I,I,SI)	(,A,U,MI)
*D*_18_	(MI,I,VI,I)	(SI,MI,I,MI)	(VI,A,SU,A)	(MI,MI,SU,A)	(MI,A,U,SI)	(I,MI,U,SU)

In accordance with Eq. (S6) (or Eq. (S11)), an A-SVN-DM is estimated (taking $\theta \left(\beta \right)=e^{\frac{1-\beta }{\beta }},\psi \left(\beta^{\prime }\right)=1-e^{-\frac{\beta^{\prime }}{1-\beta^{\prime }}}\text{,}$ where $\beta \in \left(0,1],\beta^{\prime }\in [0,1\right)\text{,}$ and shown in Table 6.

**Table 6:** A-SVN-DM for LiBs’ manufacturing plant location.

	*F*_1_	*F*_2_	*F*_3_	*F*_4_	*F*_5_	*F*_6_
*D*_1_	(0.225, 0.744, 0.700)	(0.243, 0.755, 0.705)	(0.398, 0.493, 0.502)	(0.421, 0.540, 0.539)	(0.361, 0.578, 0.556)	(0.469, 0.406, 0.442)
*D*_2_	(0.219, 0.777, 0.727)	(0.198, 0.797, 0.747)	(0.432, 0.485, 0.495)	(0.405, 0.554, 0.554)	(0.330, 0.661, 0.624)	(0.428, 0.483, 0.498)
*D*_3_	(0.374, 0.587, 0.565)	(0.209, 0.807, 0.757)	(0.439, 0.634, 0.506)	(0.395, 0.580, 0.563)	(0.352, 0.586, 0.564)	(0.337, 0.661, 0.617)
*D*_4_	(0.381, 0.545, 0.539)	(0.497, 0.440, 0.438)	(0.414, 0.531, 0.529)	(0.243, 0.755, 0.705)	(0.341, 0.634, 0.595)	(0.322, 0.665, 0.631)
*D*_5_	(0.512, 0.340, 0.384)	(0.672, 0.187, 0.238)	(0.392, 0.553, 0.549)	(0.461, 0.360, 0.391)	(0.600, 0.281, 0.333)	(0.672, 0.187, 0.238)
*D*_6_	(0.638, 0.233, 0.285)	(0.713, 0.158, 0.208)	(0.524, 0.335, 0.374)	(0.670, 0.187, 0.239)	(0.623, 0.222, 0.273)	(0.581, 0.375, 0.391)
*D*_7_	(0.379, 0.507, 0.517)	(0.196, 0.798, 0.748)	(0.526, 0.406, 0.442)	(0.424, 0.491, 0.501)	(0.277, 0.657, 0.629)	(0.350, 0.570, 0.566)
*D*_8_	(0.285, 0.696, 0.645)	(0.233, 0.757, 0.714)	(0.326, 0.557, 0.561)	(0.344, 0.619, 0.592)	(0.317, 0.627, 0.595)	(0.388, 0.579, 0.570)
*D*_9_	(0.240, 0.758, 0.708)	(0.198, 0.797, 0.747)	(0.454, 0.490, 0.493)	(0.199, 0.808, 0.766)	(0.187, 0.722, 0.708)	(0.415, 0.510, 0.509)
*D*_10_	(0.363, 0.602, 0.575)	(0.221, 0.776, 0.726)	(0.330, 0.678, 0.640)	(0.302, 0.649, 0.630)	(0.185, 0.723, 0.709)	(0.360, 0.592, 0.578)
*D*_11_	(0.524, 0.303, 0.345)	(0.685, 0.197, 0.219)	(0.403, 0.511, 0.520)	(0.414, 0.468, 0.488)	(0.482, 0.426, 0.451)	(0.576, 0.372, 0.369)
*D*_12_	(0.596, 0.233, 0.255)	(0.688, 0.194, 0.245)	(0.441, 0.504, 0.505)	(0.504, 0.403, 0.413)	(0.679, 0.214, 0.267)	(0.728, 0.199, 0.214)
*D*_13_	(0.638, 0.203, 0.229)	(0.670, 0.207, 0.259)	(0.430, 0.457, 0.474)	(0.492, 0.387, 0.423)	(0.503, 0.405, 0.433)	(0.492, 0.379, 0.400)
*D*_14_	(0.536, 0.364, 0.404)	(0.747, 0.179, 0.199)	(0.543, 0.288, 0.331)	(0.511, 0.355, 0.384)	(0.512, 0.416, 0.441)	(0.557, 0.354, 0.386)
*D*_15_	(0.584, 0.318, 0.362)	(0.668, 0.184, 0.209)	(0.537, 0.347, 0.385)	(0.518, 0.418, 0.435)	(0.522, 0.366, 0.398)	(0.568, 0.356, 0.381)
*D*_16_	(0.524, 0.335, 0.374)	(0.577, 0.254, 0.307)	(0.568, 0.298, 0.339)	(0.490, 0.447, 0.461)	(0.500, 0.384, 0.393)	(0.648, 0.259, 0.306)
*D*_17_	(0.466, 0.392, 0.419)	(0.632, 0.290, 0.333)	(0.447, 0.488, 0.500)	(0.410, 0.480, 0.491)	(0.541, 0.315, 0.361)	(0.574, 0.335, 0.371)
*D*_18_	(0.706, 0.198, 0.231)	(0.679, 0.181, 0.232)	(0.493, 0.449, 0.441)	(0.504, 0.326, 0.359)	(0.487, 0.417, 0.446)	(0.427, 0.392, 0.415)

To obtain the objective weight by MEREC, we normalize the A-SVN-DM and the find the score matrix using Eqs (4), (5). Then, we compute the overall performance values of options using Eq. (6), and is estimated as $\vartheta_{1}$ = 0.360, $\vartheta_{2}$ = 0.272, $\vartheta_{3}$ = 0.48, $\vartheta_{4}$ = 0.424, $\vartheta_{5}$ = 0.384, and $\vartheta_{6}$ = 0.406. Applying Eq. (7), we derive the LiB alternatives' overall performance $\left(\vartheta_{ij}^{\prime }\right)$ by eliminating each criterion which is shown in Table 7. Using Eq. (8), we compute the sum of deviation and hence, we estimate the objective weights of criteria for the LiBs’ manufacturing plant location and are given in the last column of Table 7.

**Table 7:** The objective weight of criteria using MEREC.

Criteria	$\left(\vartheta_{ij}^{\prime }\right)$ values						*Abd*_*j*_	$w_{j}^{o}$
	*F*_1_	*F*_2_	*F*_3_	*F*_4_	*F*_5_	*F*_6_		
*D*_1_	0.348	0.259	0.461	0.402	0.364	0.377	0.118	0.0606
*D*_2_	0.349	0.261	0.460	0.403	0.367	0.382	0.106	0.0545
*D*_3_	0.339	0.262	0.464	0.404	0.364	0.390	0.106	0.0547
*D*_4_	0.338	0.239	0.462	0.413	0.366	0.391	0.121	0.0622
*D*_5_	0.339	0.260	0.454	0.403	0.368	0.396	0.109	0.0562
*D*_6_	0.346	0.262	0.466	0.413	0.371	0.388	0.084	0.0432
*D*_7_	0.336	0.262	0.455	0.400	0.368	0.387	0.121	0.0624
*D*_8_	0.345	0.259	0.465	0.406	0.366	0.386	0.100	0.0514
*D*_9_	0.348	0.261	0.460	0.415	0.373	0.383	0.089	0.0456
*D*_10_	0.340	0.260	0.469	0.408	0.373	0.388	0.091	0.0467
*D*_11_	0.341	0.260	0.456	0.397	0.360	0.388	0.126	0.0651
*D*_12_	0.346	0.260	0.458	0.402	0.372	0.397	0.094	0.0486
*D*_13_	0.348	0.259	0.459	0.402	0.361	0.385	0.114	0.0587
*D*_14_	0.339	0.262	0.468	0.404	0.361	0.388	0.107	0.0551
*D*_15_	0.342	0.260	0.466	0.402	0.363	0.388	0.108	0.0555
*D*_16_	0.340	0.255	0.468	0.400	0.362	0.393	0.110	0.0568
*D*_17_	0.336	0.255	0.458	0.397	0.365	0.389	0.128	0.0662
*D*_18_	0.349	0.260	0.462	0.405	0.360	0.383	0.109	0.0564

According to SVN-SWARA (using Eq. (10)-Eq. (12) and Table 7, the subjective weight of criteria are estimated and mentioned in Table 8, Table 9.Thus, the subjective weights of criteria are presented as

**Table 8:** Aggregated performance values and score values of criteria.

Criteria	*e*_1_	*e*_2_	*e*_3_	*e*_4_	Aggregated SVNNs	Score values
*D*_1_	A	SI	SU	A	(0.417, 0.473, 0.485)	0.514
*D*_2_	A	A	SI	SU	(0.451, 0.517, 0.514)	0.527
*D*_3_	SI	SU	U	SU	(0.342, 0.629, 0.594)	0.627
*D*_4_	SU	A	U	SI	(0.428, 0.483, 0.498)	0.518
*D*_5_	U	U	SU	A	(0.363, 0.620, 0.593)	0.383
*D*_6_	A	SI	I	SU	(0.455, 0.465, 0.481)	0.503
*D*_7_	SU	A	I	SI	(0.517, 0.387, 0.420)	0.430
*D*_8_	SI	A	U	SU	(0.354, 0.581, 0.563)	0.597
*D*_9_	SU	A	SU	A	(0.393, 0.541, 0.528)	0.559
*D*_10_	I	SU	A	A	(0.444, 0.505, 0.502)	0.521
*D*_11_	U	SU	SI	SI	(0.471, 0.455, 0.478)	0.513
*D*_12_	SU	A	SI	I	(0.544, 0.362, 0.397)	0.595
*D*_13_	SI	SU	U	SU	(0.342, 0.629, 0.594)	0.373
*D*_14_	I	SL	SU	A	(0.468, 0.470, 0.480)	0.506
*D*_15_	SI	SI	U	SU	(0.368, 0.522, 0.526)	0.440
*D*_16_	SU	I	SU	A	(0.424, 0.439, 0.453)	0.510
*D*_17_	U	SI	SU	SU	(0.352, 0.548, 0.540)	0.422
*D*_18_	SU	SU	A	A	(0.404, 0.555, 0.538)	0.437

**Table 9:** Subjective weight of criteria using the SVN-SWARA.

Criteria	Score values	Comparative significance	Coefficient	Initial weight	$w_{j}^{s}$
*D*_3_	0.627	–	1.000	1.000	0.0629
*D*_8_	0.597	0.030	1.030	0.9709	0.0611
*D*_12_	0.595	0.002	1.002	0.9690	0.0609
*D*_9_	0.559	0.036	1.036	0.9353	0.0588
*D*_2_	0.527	0.032	1.032	0.9063	0.0570
*D*_10_	0.521	0.006	1.006	0.9009	0.0567
*D*_4_	0.518	0.003	1.003	0.8982	0.0565
*D*_1_	0.514	0.004	1.004	0.8946	0.0563
*D*_11_	0.513	0.001	1.001	0.8937	0.0562
*D*_16_	0.510	0.003	1.003	0.8910	0.0560
*D*_14_	0.506	0.004	1.004	0.8875	0.0558
*D*_6_	0.503	0.003	1.003	0.8848	0.0556
*D*_15_	0.440	0.063	1.063	0.8324	0.0523
*D*_18_	0.437	0.003	1.003	0.8299	0.0522
*D*_7_	0.430	0.007	1.007	0.8241	0.0518
*D*_17_	0.422	0.008	1.008	0.8176	0.0514
*D*_5_	0.383	0.039	1.039	0.7869	0.0495
*D*_13_	0.373	0.010	1.010	0.7791	0.0490

$w_{j}^{s}$ (0.0563, 0.0570, 0.0629, 0.0565, 0.0495, 0.0556, 0.0518, 0.0611, 0.0588, 0.0567, 0.0562, 0.0609,0.0490, 0.0558, 0.0523, 0.0560, 0.0514, 0.0522).

**Fig. 3:**
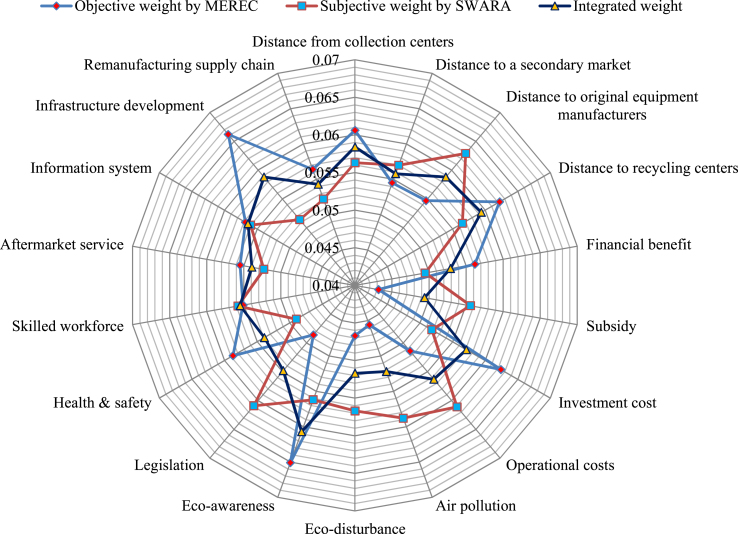
Weight of criteria for the LiBs' manufacturing plant location selection.

The final weights of criteria (τ=0.5) are presented using Eq. (13) (graphically shown in Fig. 3).

*w*_j_ = (0.0584, 0.0558, 0.0588, 0.0594, 0.0529, 0.0494, 0.0571, 0.0563, 0.0522, 0.0517, 0.0607, 0.0548, 0.0539, 0.0555, 0.0539, 0.0564, 0.0588, 0.0543).

Here, Fig. 3 spectacles the weight values of diverse criteria for LiBs’ manufacturing plant location concerning the goal. Eco-awareness (*D*_11_), with a weight of value 0.0607 has come out to be the most important parameter in the manufacturing plant locations selection for LiB. Distance to recycling centers (*D*_4_) is the second most significant criterion for the manufacturing plant locations selection for LiB with 0.0594. Infrastructure development (*D*_17_) and distance to original equipment manufacturers (*D*_3_) have third with a significance value 0.0588, distance from collection centers (*D*_1_) has the fourth rank with a weight 0.0584, investment cost (*D*_7_) with a weight 0.0571 has the fifth significant criteria to the manufacturing plant locations selection for LiB and others are considered crucial criteria to the manufacturing plant locations selection for LiBs.

Using Eqs (14), (16) and Table 5, the linear normalization and vector normalization values for LiBs’ manufacturing plant location are estimated and shown in Table 10, Table 11.

**Table 10:** Linear normalization matrix for each option.

	*F*_1_	*F*_2_	*F*_3_	*F*_4_	*F*_5_	*F*_6_
*P*_1_	(0.0375, 0.8033, 0.7682)	(0.0440, 0.8126, 0.7722)	(0.1195, 0.5927, 0.6002)	(0.1347, 0.6337, 0.6328)	(0.0982, 0.6669, 0.6480)	(0.1677, 0.5130, 0.5469)
*P*_2_	(0.0377, 0.8212, 0.7794)	(0.0308, 0.8374, 0.7959)	(0.1491, 0.5678, 0.5766)	(0.1304, 0.6305, 0.6305)	(0.0864, 0.7237, 0.6912)	(0.1462, 0.5657, 0.5799)
*P*_3_	(0.1118, 0.6579, 0.6386)	(0.0343, 0.8451, 0.8035)	(0.1549, 0.6988, 0.5860)	(0.1247, 0.6518, 0.6372)	(0.0989, 0.6570, 0.6376)	(0.0905, 0.7225, 0.6845)
*P*_4_	(0.1093, 0.6387, 0.6334)	(0.1892, 0.5453, 0.5431)	(0.1296, 0.6264, 0.6249)	(0.0439, 0.8127, 0.7723)	(0.0871, 0.7136, 0.6816)	(0.0779, 0.7398, 0.7115)
*P*_5_	(0.2035, 0.4455, 0.4880)	(0.3624, 0.2853, 0.3409)	(0.1174, 0.6414, 0.6382)	(0.1638, 0.4656, 0.4947)	(0.2846, 0.3865, 0.4385)	(0.3624, 0.2853, 0.3409)
*P*_6_	(0.3353, 0.3205, 0.3743)	(0.4257, 0.2360, 0.2931)	(0.2218, 0.4254, 0.4637)	(0.3724, 0.2695, 0.3267)	(0.3188, 0.3079, 0.3626)	(0.2749, 0.4643, 0.4794)
*P*_7_	(0.1142, 0.5877, 0.5966)	(0.0303, 0.8383, 0.7968)	(0.2245, 0.4932, 0.5278)	(0.1440, 0.5727, 0.5820)	(0.0607, 0.7198, 0.6956)	(0.0974, 0.6440, 0.6400)
*P*_8_	(0.0613, 0.7629, 0.7213)	(0.0409, 0.8126, 0.7778)	(0.0806, 0.6464, 0.6495)	(0.0898, 0.6989, 0.6764)	(0.0758, 0.7061, 0.6788)	(0.1147, 0.6652, 0.6572)
*P*_9_	(0.0458, 0.8031, 0.7608)	(0.0312, 0.8356, 0.7936)	(0.1669, 0.5689, 0.5714)	(0.0314, 0.8444, 0.8099)	(0.0278, 0.7727, 0.7605)	(0.1392, 0.5866, 0.5859)
*P*_10_	(0.1018, 0.6801, 0.6568)	(0.0373, 0.8246, 0.7836)	(0.0841, 0.7443, 0.7127)	(0.0700, 0.7195, 0.7041)	(0.0263, 0.7817, 0.7700)	(0.1002, 0.6716, 0.6590)
*P*_11_	(0.2153, 0.4051, 0.4473)	(0.3807, 0.2930, 0.3173)	(0.1254, 0.6018, 0.6097)	(0.1323, 0.5636, 0.5814)	(0.1815, 0.5248, 0.5473)	(0.2628, 0.4734, 0.4706)
*P*_12_	(0.2874, 0.3249, 0.3482)	(0.3907, 0.2818, 0.3378)	(0.1534, 0.5892, 0.5904)	(0.2024, 0.4961, 0.5054)	(0.3796, 0.3046, 0.3611)	(0.4409, 0.2879, 0.3045)
*P*_13_	(0.3189, 0.3097, 0.3378)	(0.3547, 0.3142, 0.3708)	(0.1398, 0.5621, 0.5778)	(0.1841, 0.4980, 0.5314)	(0.1930, 0.5145, 0.5401)	(0.1841, 0.4896, 0.5094)
*P*_14_	(0.2348, 0.4504, 0.4887)	(0.4750, 0.2573, 0.2795)	(0.2408, 0.3739, 0.4182)	(0.2128, 0.4410, 0.4698)	(0.2133, 0.5002, 0.5237)	(0.2541, 0.4405, 0.4713)
*P*_15_	(0.2716, 0.4194, 0.4625)	(0.3614, 0.2768, 0.3055)	(0.2276, 0.4479, 0.4851)	(0.2111, 0.5156, 0.5320)	(0.2144, 0.4666, 0.4972)	(0.2560, 0.4571, 0.4808)
*P*_16_	(0.1996, 0.4682, 0.5055)	(0.2449, 0.3859, 0.4402)	(0.2372, 0.4313, 0.4722)	(0.1733, 0.5714, 0.5840)	(0.1812, 0.5145, 0.5227)	(0.3154, 0.3919, 0.4395)
*P*_17_	(0.1514, 0.5341, 0.5590)	(0.2888, 0.4369, 0.4786)	(0.1385, 0.6189, 0.6283)	(0.1161, 0.6115, 0.6210)	(0.2070, 0.4611, 0.5053)	(0.2349, 0.4808, 0.5146)
*P*_18_	(0.4074, 0.2923, 0.3291)	(0.3748, 0.2731, 0.3305)	(0.1904, 0.5442, 0.5377)	(0.1995, 0.4267, 0.4600)	(0.1860, 0.5147, 0.5421)	(0.1417, 0.4909, 0.5132)

**Table 11:** Vector normalization matrix for each LiB manufacturing plant location.

	*F*_1_	*F*_2_	*F*_3_	*F*_4_	*F*_5_	*F*_6_
*P*_1_	(0.2518, 0.5064, 0.4909)	(0.2723, 0.5143, 0.4943)	(0.4458, 0.3357, 0.3517)	(0.4727, 0.3675, 0.3777)	(0.4049, 0.3937, 0.3900)	(0.5256, 0.2762, 0.3101)
*P*_2_	(0.2565, 0.4964, 0.4820)	(0.2318, 0.5090, 0.4951)	(0.5057, 0.3096, 0.3279)	(0.4737, 0.3541, 0.3675)	(0.3867, 0.4223, 0.4134)	(0.5008, 0.3082, 0.3302)
*P*_3_	(0.4264, 0.3701, 0.3840)	(0.2376, 0.5091, 0.5145)	(0.5004, 0.3997, 0.3441)	(0.4500, 0.3657, 0.3829)	(0.4015, 0.3694, 0.3832)	(0.3842, 0.4170, 0.4195)
*P*_4_	(0.4149, 0.3687, 0.3800)	(0.5416, 0.2976, 0.3087)	(0.4508, 0.3591, 0.3732)	(0.2646, 0.5107, 0.4970)	(0.3711, 0.4283, 0.4197)	(0.3513, 0.4497, 0.4448)
*P*_5_	(0.3722, 0.4061, 0.4224)	(0.4886, 0.2240, 0.2617)	(0.2849, 0.6606, 0.6045)	(0.3351, 0.4307, 0.4302)	(0.4366, 0.3359, 0.3662)	(0.4886, 0.2240, 0.2617)
*P*_6_	(0.4149, 0.3617, 0.3842)	(0.4636, 0.2446, 0.2811)	(0.3408, 0.5194, 0.5051)	(0.4358, 0.2897, 0.3229)	(0.4052, 0.3437, 0.3689)	(0.3777, 0.5809, 0.5271)
*P*_7_	(0.4135, 0.3537, 0.3667)	(0.2144, 0.5566, 0.5304)	(0.5749, 0.2828, 0.3135)	(0.4633, 0.3423, 0.3552)	(0.3028, 0.4582, 0.4460)	(0.3823, 0.3975, 0.4010)
*P*_8_	(0.3646, 0.4419, 0.4284)	(0.2982, 0.4809, 0.4739)	(0.4174, 0.3539, 0.3722)	(0.4402, 0.3929, 0.3930)	(0.4048, 0.3983, 0.3949)	(0.4963, 0.3678, 0.3781)
*P*_9_	(0.3233, 0.4464, 0.4350)	(0.2670, 0.4693, 0.4588)	(0.6121, 0.2888, 0.3030)	(0.2682, 0.4756, 0.4708)	(0.2524, 0.4252, 0.4348)	(0.5602, 0.3002, 0.3127)
*P*_10_	(0.4917, 0.3652, 0.3637)	(0.2991, 0.4705, 0.4588)	(0.4476, 0.4112, 0.4049)	(0.4090, 0.3933, 0.3985)	(0.2513, 0.4386, 0.4483)	(0.4879, 0.3592, 0.3653)
*P*_11_	(0.4088, 0.3137, 0.3426)	(0.5347, 0.2044, 0.2176)	(0.3144, 0.5295, 0.5160)	(0.3229, 0.4855, 0.4845)	(0.3764, 0.4418, 0.4473)	(0.4496, 0.3855, 0.3663)
*P*_12_	(0.3959, 0.3023, 0.3118)	(0.4570, 0.2514, 0.2998)	(0.2926, 0.6539, 0.6181)	(0.3347, 0.5232, 0.5053)	(0.4510, 0.2781, 0.3268)	(0.4831, 0.2584, 0.2620)
*P*_13_	(0.4784, 0.2345, 0.2449)	(0.5025, 0.2391, 0.2781)	(0.3228, 0.5274, 0.5083)	(0.3688, 0.4473, 0.4536)	(0.3772, 0.4676, 0.4638)	(0.3688, 0.4371, 0.4282)
*P*_14_	(0.3817, 0.4444, 0.4506)	(0.5317, 0.2187, 0.2220)	(0.3863, 0.3510, 0.3699)	(0.3639, 0.4327, 0.4287)	(0.3644, 0.5075, 0.4918)	(0.3965, 0.4321, 0.4304)
*P*_15_	(0.4195, 0.3827, 0.4006)	(0.4796, 0.2212, 0.4006)	(0.3857, 0.4174, 0.4006)	(0.3719, 0.5026, 0.4006)	(0.3748, 0.4405, 0.4006)	(0.4079, 0.4287, 0.4006)
*P*_16_	(0.3861, 0.4067, 0.4160)	(0.4252, 0.3078, 0.3409)	(0.4189, 0.3613, 0.3771)	(0.3609, 0.5416, 0.5121)	(0.3687, 0.4658, 0.4366)	(0.4780, 0.3147, 0.3401)
*P*_17_	(0.3679, 0.4091, 0.4379)	(0.4983, 0.3031, 0.3472)	(0.3524, 0.5098, 0.5214)	(0.3237, 0.5007, 0.5124)	(0.4268, 0.3285, 0.3765)	(0.4530, 0.3497, 0.3869)
*P*_18_	(0.5152, 0.2354, 0.2580)	(0.4959, 0.2152, 0.2594)	(0.3598, 0.5339, 0.4926)	(0.3680, 0.3874, 0.4011)	(0.3558, 0.4960, 0.4980)	(0.3118, 0.4660, 0.4633)

The subordinate UDs of the CCM, UCM and ICM are computed by means of Eq. (17)-Eq. (19) (taking $\theta \left(\beta \right)=e^{\frac{1-\beta }{\beta }},\psi \left(\beta^{\prime }\right)=1-e^{-\frac{\beta^{\prime }}{1-\beta^{\prime }}}\text{,}$ where $\beta \in \left(0,1],\beta^{\prime }\in [0,1\right)\text{,}$ and portrayed in Table 12. Corresponding to Eq. (20), the normalized subordinate UDs are estimated and stated in Table 13.

**Table 12:** Results of CCM, UCM and ICM for each LiB manufacturing plant location.

Option	CCM		UCM		ICM	
	$C_{1}$	$S\left(C_{1}\right)$	$C_{2}$	$S\left(C_{2}\right)$	$C_{3}$	$S\left(C_{3}\right)$
*F*_1_	(0.186, 0.513, 0.531)	0.381	(0.047, 0.956, 0.895)	0.065	(0.396, 0.373, 0.383)	0.547
*F*_2_	(0.243, 0.463, 0.490)	0.430	(0.038, 0.967, 0.930)	0.047	(0.414, 0.325, 0.354)	0.578
*F*_3_	(0.162, 0.558, 0.567)	0.346	(0.057, 0.945, 0.890)	0.074	(0.420, 0.418, 0.417)	0.528
*F*_4_	(0.154, 0.565, 0.577)	0.337	(0.055, 0.946, 0.881)	0.076	(0.383, 0.425, 0.425)	0.511
*F*_5_	(0.167, 0.550, 0.567)	0.350	(0.041, 0.960, 0.908)	0.0575	(0.376, 0.409, 0.415)	0.517
*F*_6_	(0.209, 0.505, 0.522)	0.394	(0.038, 0.964, 0.899)	0.0583	(0.443, 0.365, 0.373)	0.568

**Table 13:** Normalized CCM, UCM and ICM degrees and OUDs of the LiBs’ manufacturing plant locations.

Option	CCM		UCM		ICM		$U_{i}$	Final Ranking
	$C_{1}^{\left(N\right)}$	$\rho_{1}$	$C_{2}^{\left(N\right)}$	$\rho_{2}$	$C_{3}^{\left(N\right)}$	$\rho_{3}$		
*F*_1_	0.415	3	0.418	4	0.412	3	0.6389	3
*F*_2_	0.469	1	0.301	1	0.435	1	0.7579	1
*F*_3_	0.377	5	0.472	5	0.398	4	0.5747	5
*F*_4_	0.368	6	0.487	6	0.385	6	0.5338	6
*F*_5_	0.382	4	0.368	2	0.390	5	0.6277	4
*F*_6_	0.430	2	0.373	3	0.428	2	0.6900	2
Weight of aggregation model	w1=13		w2=13		w3=13			

From Eq. (21), the OUD of each location is computed and shown in Table 13. Regardless of assuming w1=w2=w3=13, the weights can be selected according to DEs' preferences on the basis of poor performances of alternatives or their widespread accomplishment. Hence, the LiBs’ manufacturing plant location *F*_2_ has the highest utility degree of the appropriateness of options.

### 5.1 Sensitivity investigation

**Fig. 4:**
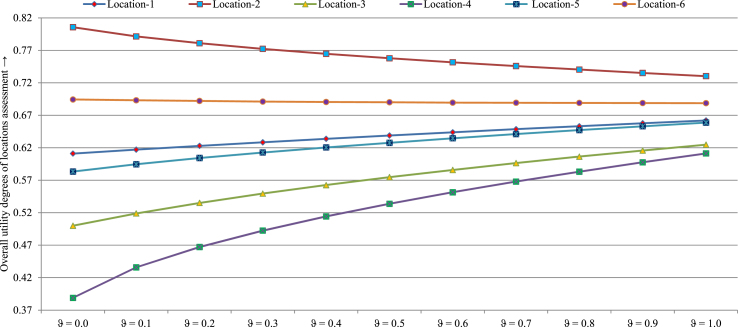
Sensitivity outcomes of the *UD*$y_{i}^{*}\text{,}$ values over the utility parameter *ϑ*

Here, we consider the variation of factor ϑ in the proposed DNMA model and further analyze the consequence of objective and subjective weighting for criteria. For this purpose, the following two cases are discussed:

Case I: Varying the values of *ϑ* is used to analyze the sensitivity of the presented method. Table 14 and Fig. 4 show the obtained results of the manufacturing location options for varied values of factor *ϑ*. By means of the assessment, we get same ranking order $F_{2}≻F_{6}≻F_{1}≻F_{5}≻F_{3}≻F_{4}$ for each factor *ϑ* values, which implies *F*_2_ is the most suitable location, whereas the *F*_4_ is the least suitable location for each parameter *ϑ* value. As a result, it is observed that the proposed framework have adequate stability with several values of parameter.

**Table 14:** Obtained outcomes by SVN-MEREC-SWARA-DNMA using diverse values of *ϑ*

$d_{i}^{*}$	*F*_1_	*F*_2_	*F*_3_	*F*_4_	*F*_5_	*F*_6_	Ranking order
*ϑ* = 0.0	0.6111	0.8056	0.5000	0.3889	0.5833	0.6944	$F_{2}≻F_{6}≻F_{1}≻F_{5}≻F_{3}≻F_{4}$
*ϑ* = 0.1	0.6172	0.7914	0.5189	0.4359	0.5946	0.6931	$F_{2}≻F_{6}≻F_{1}≻F_{5}≻F_{3}≻F_{4}$
*ϑ* = 0.2	0.6229	0.7810	0.5351	0.4672	0.6042	0.6921	$F_{2}≻F_{6}≻F_{1}≻F_{5}≻F_{3}≻F_{4}$
*ϑ* = 0.3	0.6284	0.7723	0.5495	0.4925	0.6127	0.6912	$F_{2}≻F_{6}≻F_{1}≻F_{5}≻F_{3}≻F_{4}$
*ϑ* = 0.4	0.6338	0.7647	0.5626	0.5143	0.6205	0.6905	$F_{2}≻F_{6}≻F_{1}≻F_{5}≻F_{3}≻F_{4}$
*ϑ* = 0.5	0.6389	0.7579	0.5747	0.5338	0.6277	0.6900	$F_{2}≻F_{6}≻F_{1}≻F_{5}≻F_{3}≻F_{4}$
*ϑ* = 0.6	0.6438	0.7516	0.5859	0.5515	0.6346	0.6895	$F_{2}≻F_{6}≻F_{1}≻F_{5}≻F_{3}≻F_{4}$
*ϑ* = 0.7	0.6486	0.7458	0.5964	0.5679	0.6411	0.6892	$F_{2}≻F_{6}≻F_{1}≻F_{5}≻F_{3}≻F_{4}$
*ϑ* = 0.8	0.6532	0.7404	0.6064	0.5832	0.6472	0.6890	$F_{2}≻F_{6}≻F_{1}≻F_{5}≻F_{3}≻F_{4}$
*ϑ* = 0.9	0.6577	0.7352	0.6158	0.5976	0.6531	0.6888	$F_{2}≻F_{6}≻F_{1}≻F_{5}≻F_{3}≻F_{4}$
*ϑ* = 1.0	0.6621	0.7303	0.6248	0.6113	0.6588	0.6887	$F_{2}≻F_{6}≻F_{1}≻F_{5}≻F_{3}≻F_{4}$

**Fig. 5:**
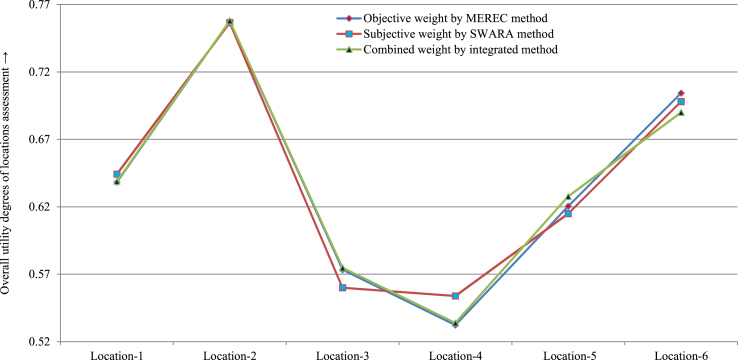
Sensitivity analysis of LiBs' manufacturing plant location with different weighting processes.

Case II: In this case, firstly we have taken only objective weights. Then the priority results have been assessed using objective weighting instead of the SVN-MEREC-SWARA tool. Using SVN-MEREC, the OUDs and preferences are given in Table 15 and Fig. 5. The OUDs of locations are *F*_1_ = 0*.*6382, *F*_2_ = 0*.*7577, *F*_3_ = 0*.*5733, *F*_4_ = 0*.*5323, *F*_5_ = 0*.*6204 and *F*_6_ = 0*.*7043, and the ranking order of LiBs’ manufacturing plant location are given in the following form $F_{2}≻F_{6}≻F_{1}≻F_{5}≻F_{3}≻F_{4}\text{.}$ Thus, it can be concluded that using the different values of parameter will enhance the solidity of the SVN-MEREC-SWARA-DNMA methodology.

**Table 15:** Subordinate *UD* of LiBs’ manufacturing plant location over diverse weighting processes.

Weighting process	Subordinate UDs of LiBs' manufacturing plant location options						Ranking order
	*F*_1_	*F*_2_	*F*_3_	*F*_4_	*F*_5_	*F*_6_	
SVN-MEREC	0.6382	0.7577	0.5733	0.5323	0.6204	0.7043	$F_{2}≻F_{6}≻F_{1}≻F_{5}≻F_{3}≻F_{4}$
SVN-SWARA	0.6442	0.7565	0.5600	0.5538	0.6149	0.6980	$F_{2}≻F_{6}≻F_{1}≻F_{5}≻F_{3}≻F_{4}$
Integrated method	0.6389	0.7579	0.5747	0.5338	0.6277	0.6900	$F_{2}≻F_{6}≻F_{1}≻F_{5}≻F_{3}≻F_{4}$

### 5.2 Comparative study

Next, we compare the developed and extant MCDM methods to confirm the efficiency of introduced methodology. The selected methods are SVN-COPRAS [80], SVN-MULTIMOORA [81] and SVN-WASPAS [82].

#### 5.2.1 SVN-COPRAS model

**Steps 1–4:** Same as SVN-MEREC-SWARA-DNMA model.
**Steps 5:** As the abovementioned example consists of benefit and cost types of criteria, so, the assessment value of each plant location alternative is computed to maximize the benefit and minimize the cost preferences, as $\chi_{i}=\overset{n_{1}}{\underset{j=1}{⊕}}w_{j}\;o_{ij}=\left(1-\prod_{j=1}^{n_{1}}{\left(1-tm_{ij}\right)}^{w_{j}},\prod_{j=1}^{n_{1}}{\left(im_{ij}\right)}^{w_{j}},\prod_{j=1}^{n_{1}}{\left(fm_{ij}\right)}^{w_{j}}\right),\;\forall \;i$ and $I_{i}=\overset{n}{\underset{j=n_{1}+1}{⊕}}w_{j}\;o_{ij}=\left(1-\prod_{j=n_{1}+1}^{n}{\left(1-tm_{ij}\right)}^{w_{j}},\prod_{j=n_{1}+1}^{n}{\left(im_{ij}\right)}^{w_{j}},\prod_{j=n_{1}+1}^{n}{\left(fm_{ij}\right)}^{w_{j}}\right),\;\forall \;i\text{,}$ respectively.
**Step 6:** Find the “relative degree (*RD*)” of the plant location alternative.

The *RD* Θ of *i*^th^ option is computed by

(22) $$z_{i}=\varphi \;S\left(\chi_{i}\right)+\left(1-\varphi \right)\;\frac{\min_{i}\;S\left(I_{i}\right)\;\sum_{i=1}^{m}S\left(I_{i}\right)}{S\left(I_{i}\right)\;\sum_{i=1}^{m}\frac{\min_{i}\;S\left(I_{i}\right)}{S\left(I_{i}\right)}},\;\forall \;i\text{.}$$

**Step 7:** Estimate the priority and UDs of each alternative.

In accordance with *RD*, the priority degree of each plant location alternative is determined. Using Eq. (23), the utility degree is computed as

(23) $$\psi_{i}=\frac{z_{i}}{z_{\max}}\times 100\%,\;\forall \;i\text{,}$$

wherein $0\leq tm_{C}\left(x\right)+im_{C}\left(x\right)+fm_{C}\left(x\right)\leq 3,\forall x\in \Theta \text{.}$ and $\left(tm_{C}\left(x\right),im_{C}\left(x\right),fm_{C}\left(x\right)\right)$ determines the *RDs* of plant location alternative.

Step 9: End.

The overall computational steps of SVN-COPRAS methodology are given in Table 16. From Table 16, the ranking order of the LiBs' manufacturing plant locations is $F_{2}≻F_{1}≻F_{5}≻F_{6}≻F_{4}≻F_{3}\text{.}$ Thus, the option *F*_2_ is the optimum LiBs’ manufacturing plant location.

**Table 16:** Results of SVN-COPRAS model.

Location	$\chi_{i}$	$S\left(\chi_{i}\right)$	$I_{i}$	$S\left(I_{i}\right)$	$z_{i}$	Ordering
*F*_1_	(0.155, 0.597, 0.628)	0.310	(0.036, 0.859, 0.846)	0.110	0.4358	2
*F*_2_	(0.223, 0.516, 0.557)	0.383	(0.026, 0.897, 0.879)	0.083	0.5489	1
*F*_3_	(0.103, 0.696, 0.711)	0.232	(0.066, 0.802, 0.797)	0.156	0.3211	6
*F*_4_	(0.114, 0.668, 0.689)	0.252	(0.045, 0.846, 0.838)	0.120	0.3673	5
*F*_5_	(0.138, 0.640, 0.669)	0.277	(0.033, 0.860, 0.847)	0.109	0.4043	3
*F*_6_	(0.164, 0.620, 0.642)	0.301	(0.054, 0.815, 0.813)	0.142	0.3981	4

#### 5.2.2 SVN-WASPAS model

This method implicates the succeeding procedures:

Steps 1–4: Same as SVN-MEREC-SWARA-DNMA.

**Step 5:** Derive the “weighted sum model (WSM)” and “weighted product model (WPM)” measures for each plant location candidate by Eq. (24) and Eq. (25), respectively.

(24) $$Q_{i}^{\left(1\right)}=\overset{n}{\underset{j=1}{⊕}}\;w_{i}\;o_{ij}=\left(1-\prod_{j=1}^{n}{\left(1-t{\tilde{m}}_{ij}\right)}^{w_{j}},\prod_{j=1}^{n}{\left(i{\tilde{m}}_{ij}\right)}^{w_{j}},\prod_{j=1}^{n}{\left(f{\tilde{m}}_{ij}\right)}^{w_{j}}\right)\text{,}$$

(25) $$Q_{i}^{\left(2\right)}=\overset{n}{\underset{j=1}{⊗}}\;o_{ij}^{w_{j}}=\left(\prod_{j=1}^{n}{\left(t{\tilde{m}}_{ij}\right)}^{w_{j}},1-\prod_{j=1}^{n}{\left(1-i{\tilde{m}}_{ij}\right)}^{w_{j}},1-\prod_{j=1}^{n}{\left(1-f{\tilde{m}}_{ij}\right)}^{w_{j}}\right)\text{.}$$

**Step 6:** Derive the measure of WASPAS by using

(26) $$Q_{i}=\tau \;Q_{i}^{\left(1\right)}+\left(1-\tau \right)\;Q_{i}^{\left(2\right)}\text{.}$$

**Step 7:** Rank the alternative(s) as per the values of *Q*_*i*_*.*

Using Eq. (24)-Eq. (26), the procedural steps of SVN-WASPAS method are computed and presented in Table 17. Hence, the priority of LiBs' manufacturing plant location is $F_{2}≻F_{6}≻F_{1}≻F_{3}≻F_{5}≻F_{4}\text{.}$ Thus, *F*_2_ is the most suitable LiBs’ manufacturing plant location alternative from sustainability perspective.

**Table 17:** Results of SVN-WASPAS model.

Location	WSM measure		WPM measure		*WASPAS*	Ranking
	$Q_{i}^{\left(1\right)}$	$S\left(Q_{i}^{\left(1\right)}\right)$	$Q_{i}^{\left(2\right)}$	$S\left(Q_{i}^{\left(2\right)}\right)$		
*F*_1_	(0.186, 0.513, 0.531)	0.381	(0.141, 0.583, 0.578)	0.327	0.3590	3
*F*_2_	(0.243, 0.463, 0.490)	0.430	(0.140, 0.610, 0.591)	0.313	0.3832	1
*F*_3_	(0.162, 0.558, 0.567)	0.346	(0.153, 0.577, 0.577)	0.333	0.3407	4
*F*_4_	(0.154, 0.565, 0.577)	0.337	(0.131, 0.607, 0.605)	0.307	0.3250	6
*F*_5_	(0.167, 0.550, 0.567)	0.350	(0.129, 0.596, 0.596)	0.312	0.3348	5
*F*_6_	(0.209, 0.505, 0.522)	0.394	(0.179, 0.541, 0.547)	0.364	0.3817	2

#### 5.2.3 SVN-MULTIMOORA model

The way of MULTIMOORA model is discussed as

**Steps 1–4:** Same as SVN-MEREC-SWARA-DNMA model.
**Step 5:** Use the ratio system procedure to rank the options as
**Step 5a:** Compute $\sum_{j=1}^{n}\varpi_{j}=1\text{.}$ and $SVNWA\left(\alpha_{1},\alpha_{2},...,\alpha_{n}\right)=\overset{n}{\underset{j=1}{⊕}}\left(\varpi_{j}\;\alpha_{j}\right)=\left(1-\prod_{j=1}^{n}{\left(1-tm_{j}\right)}^{\varpi_{j}},\prod_{j=1}^{n}{\left(im_{j}\right)}^{\varpi_{j}},\prod_{j=1}^{n}{\left(fm_{j}\right)}^{\varpi_{j}}\right)\text{,}$ by applying the SVNWAO as

(27) $$Y_{i}^{+}=\left(1-\prod_{j\in P_{b}}{\left(1-tm_{ij}\right)}^{w_{j}},\prod_{j\in P_{b}}{\left(im_{ij}\right)}^{w_{j}},\prod_{j\in P_{b}}{\left(fm_{ij}\right)}^{w_{j}}\right)\text{,}$$

(28) $$Y_{i}^{-}=\left(1-\prod_{j\in P_{n}}{\left(1-tm_{ij}\right)}^{w_{j}},\prod_{j\in P_{n}}{\left(im_{ij}\right)}^{w_{j}},\prod_{j\in P_{n}}{\left(fm_{ij}\right)}^{w_{j}}\right)\text{,}$$

where $D_{h}\left(\alpha_{1},\alpha_{2}\right)=$ and $\frac{1}{3n}\;\sum_{i=1}^{n}\left(\left|tm_{\alpha_{1}}\left(x_{i}\right)-tm_{\alpha_{2}}\left(x_{i}\right)\right|+\left|im_{\alpha_{1}}\left(x_{i}\right)-im_{\alpha_{2}}\left(x_{i}\right)\right|+\left|fm_{\alpha_{1}}\left(x_{i}\right)-fm_{\alpha_{2}}\left(x_{i}\right)\right|\right)\text{.}$ represent the significance of an option *F*_*i*_.

**Step 5b:** Obtain the θ:(0,1]→R and $\delta (x,x^{\prime })=\theta^{-1}(\theta (x)+\theta (x^{\prime }))\;for\;x,x^{\prime }\in (0,1]\text{.}$ as follows:

(29) $$y_{i}^{+}=S\left(Y_{i}^{+}\right)\;and\;y_{i}^{-}=S\left(Y_{i}^{-}\right)\text{.}$$

**Step 5c:** The “overall significance value” of the option is obtained as

(30) $$y_{i}=y_{i}^{+}-y_{i}^{-}\text{.}$$

**Step 6:** Utilize the reference point procedure to rank the option as
**Step 6a:** Estimate the reference point. Each coordinate value $s^{*}=\left\{s_{1}^{*},s_{2}^{*},...,s_{n}^{*}\right\}$ is a SVNN, which is defined by

(31) $$s_{j}^{*}=\left\{ \begin{array}{l} \left(\max_{i}\;tm_{ij},\min_{i}\;im_{ij},\min_{i}\;fm_{ij}\right),j\in P_{b}\\ \left(\min_{i}\;tm_{ij},\max_{i}\;im_{ij},\max_{i}\;fm_{ij}\right),j\in P_{n} \end{array}\right.\text{.}$$

**Step 6b:** Evaluate the *weighted discrimination degree (WDD)* from each option with $s_{j}^{*}$ as

(32) $$D_{ij}=w_{j}\;\left(D_{h}\left(o_{ij},s_{j}^{*}\right)\right)\text{.}$$

**Step 6c:** The maximum discrimination value is obtained as

(33) $$d_{i}=\max_{j}\;D_{ij},\forall i\text{.}$$

**Step 7:** Use the *full multiplicative form* procedure to rank the option.
**Step 7a:** Assess *A*_*i*_ and *B*_*i*_ using the SVNWG as

(34) $$A_{i}=\left(\prod_{j\in P_{b}}{\left(tm_{ij}\right)}^{w_{j}},1-\prod_{j\in P_{b}}{\left(1-im_{ij}\right)}^{w_{j}},1-\prod_{j\in P_{b}}{\left(1-fm_{ij}\right)}^{w_{j}}\right)\text{,}$$

(35) $$B_{i}=\left(\prod_{j\in P_{n}}{\left(tm_{ij}\right)}^{w_{j}},1-\prod_{j\in P_{n}}{\left(1-im_{ij}\right)}^{w_{j}},1-\prod_{j\in P_{n}}{\left(1-fm_{ij}\right)}^{w_{j}}\right)\text{.}$$

**Step 7b:** Compute $a_{i}$ and $b_{i}$ using

(36) $$a_{i}=S\left(A_{i}\right)\;and\;b_{i}=S\left(B_{i}\right)\text{.}$$

**Step 7c:** Estimate the OUD for each option as

(37) $$u_{i}=\frac{a_{i}}{b_{i}}\text{.}$$

**Step 8:** The overall assessment degree (OAD) of each option.

First, we use vector normalization to obtain the normalized scores of the ratio system, reference point and full multiplicative form denoted as $y_{i}^{*},d_{i}^{*}$ and $u_{i}^{*}\text{,}$ respectively. Then the OAD is obtained by

(38) $$O_{i}=y_{i}^{*}.\;\frac{m-\rho \left(y_{i}^{*}\right)+1}{\left(m\;\left(m+1\right)/2\right)}-d_{i}^{*}.\;\frac{\rho \left(d_{i}^{*}\right)}{\left(m\;\left(m+1\right)/2\right)}+u_{i}^{*}.\;\frac{m-\rho \left(u_{i}^{*}\right)+1}{\left(m\;\left(m+1\right)/2\right)},\forall i\text{,}$$

where $y_{i}^{*}=\frac{y_{i}}{\sqrt{\sum_{i=1}^{m}{\left(y_{i}\right)}^{2}}},d_{i}^{*}=\frac{d_{i}}{\sqrt{\sum_{i=1}^{m}{\left(d_{i}\right)}^{2}}}$ and $u_{i}^{*}=\frac{u_{i}}{\sqrt{\sum_{i=1}^{m}{\left(u_{i}\right)}^{2}}}\text{.}$ $\rho \left(y_{i}^{*}\right),\rho \left(d_{i}^{*}\right)$ and $\rho \left(u_{i}^{*}\right)$ are the ranking orders of the ratio system, reference point and full multiplicative form procedures, respectively. The optimum alternative has the highest OAD $\left(O_{i}\right)$.

**Fig. 6:**
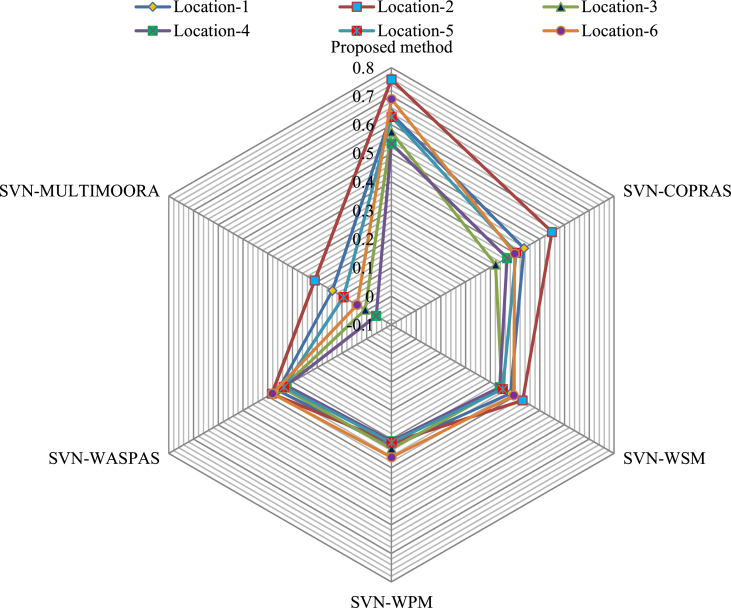
Variation of *OUDs* of LiBs' manufacturing plant location with different models.

Using Eq. (27)-Eq. (37), the ratio system, reference point and full multiplicative form models' assessment values and their respective ranking are presented in Table 18. Hence, the priority of the LiBs' manufacturing plant location is $F_{2}≻F_{1}≻F_{5}≻F_{6}≻F_{3}≻F_{4}\text{.}$ Thus, the optimal LiBs’ manufacturing plant location option is *F*_2_. Comparative results are graphically shown in Fig. 6.

**Table 18:** Results obtained by SVN-MULTIMOORA method.

Locations	Ratio system model		Reference point model		Full multiplicative form model		$O_{i}$	Final Ranking
	$y_{i}^{*}$	$\rho \left(y_{i}^{*}\right)$	$d_{i}^{*}$	$\rho \left(d_{i}^{*}\right)$	$u_{i}^{*}$	$\rho \left(u_{i}^{*}\right)$		
*F*_1_	0.440	2	0.399	3.5	0.422	2	0.139	2
*F*_2_	0.659	1	0.469	6	0.546	1	0.210	1
*F*_3_	0.168	6	0.356	1	0.309	6	0.006	5
*F*_4_	0.290	5	0.421	5	0.365	5	−0.038	6
*F*_5_	0.370	3	0.397	2	0.400	3	0.094	3
*F*_6_	0.349	4	0.399	3.5	0.368	4	0.039	4

The major advantages of the present approach are given as

• In the present model, the weights of DEs are computed with the ranking sum weighting procedure providing more accurate results, whereas in SVN-WASPAS and SVN-MULTIMOORA, the weights of DEs are computed with the score function-based procedure, and in SVN-COPRAS, the weights of DEs are randomly chosen.
• The present methodology uses an incorporated criteria weights by utilizing objective weights by SVN-MEREC and subjective weights by SVN-SWARA, therefore avoids the drawbacks of using only objective weights or subjective weights of criteria [[80], [81], [82]].
• In the proposed SVN-MEREC-SWARA-DNMA methodology, the “SVN-Archimedean-Dombi weighted aggregation operators (AOs)” are introduced in order to include higher flexibility and generality during the process of aggregation. Since “SVN-algebraic”, “SVN-Einstein” and SVN-Hamacher” AOs are particular cases of the presented AOs. Thus, the presented AOs are more significant.
• SVN-MEREC-SWARA-DNMA method utilizes three aggregation models to determine three kinds of subordinate UDs according to linear and vector normalizations. The preference ordering of the plant locations is presented based on the aggregation of subordinate ranks.
• The SVN-COPRAS tool is defined from complex proportional associations between the values of SVN-DM with the SVNWA. It suffers the following deficiencies: (a) the non-meaningfulness of preferences in mixed data settings, (b) rank reversals or preference irregularities (the preferences of options may change if an alternative is added/removed/replaced from it). Next, the SVN-WASPAS tool associates the WPM and WSM using the only aggregation information and corresponding score values to obtain the utility degree, which losses the information during the computational procedure. Remember that it is too unrealistic for the two above-mentioned benchmarks to be achieved practically. Moreover, it should be noted that the developed SVN-MEREC-SWARA-DNMA incorporates various normalization processes and aggregation functions. The OUD of the DNMA approach takes into consideration widely the subordinate UDs and the preferences of alternatives. This way, the overall preferences could be highly reliable and more realistic than the DEs.

## 6 Conclusions

This work explores the EV industry sustainability perspectives and enhances the large-scale LiB manufacturing applications in the manufacturing sectors. The comprehensive decision-making method comprising four prime aspects and eighteen assessment parameters/criteria provides an optimum one for manufacturing procedures when making a decision on plant location selection. The proposed three-way integrated methodology on SVNSs offers a straightforward and flexible optimization approach for recognizing the suitable LiB manufacturing plant location under uncertain settings. The MEREC-SWARA weighting procedure efficiently determines the objective, subjective and integrated optimal weights of EEST aspects and various assessment parameters with minimum subjectivity and biasedness. The extended SVN-DNMA approach prioritizes the LiB manufacturing plant locations with high precision. A case study delivers the presented methodology on how to find the appropriate location for a LiB manufacturing plant in the real problem. The outcomes show that location-II (*F*_2_) with an overall utility degree (0.7579) is the best location for the LiB manufacturing plant. The comparative study with extant methods and the sensitivity investigation have been revealed the high reliability and high robustness of the presented methodology for LiB manufacturing plant location selection. A transition phase to more frequent utilization of LiBs is visualized in various disciplines, comprising EVs, in the domain. Thus, this work could have high significance as the manufacturing plant location assessment problem is becoming increasingly significant.

The proposed work has some limitations, which are as follows.

• The proposed method has limitation in dealing with correlative MADM problems.
• During the assessment of LiB manufacturing plant locations, the number of decision experts contributed is limited.
• In this study, more sustainability criteria should be considered.

In future, it would be exciting to overcome the limitations of the present study. Moreover, this work can be explored in various ways. Initially, the presented methodology can be used for treating MCDM concerns with uncertainty in environmental and construction management, military applications, energy management, health and safety management, logistics, and manufacturing engineering disciplines. In further study, several other FSs can be utilized for the presented hybridized methodology, namely as rough fuzzy sets (RFSs), interval-valued hesitant fuzzy sets (IVHFSs), complex bipolar fuzzy sets (CBFSs) and complex Pythagorean fuzzy sets (CPFSs) to capture ambiguity of experts’ subjective judgments.

## Author contribution statement

Pratibha Rani; Arunodaya Raj Mishra: Conceived and designed the experiments; Wrote the paper.

Abhijit Saha: Performed the experiments.

Ibrahim M. Hezam: Analyzed and interpreted the data; Contributed reagents, materials, analysis tools or data.

Fausto Cavallaro; Ripon K. Chakrabortty: Analyzed and interpreted the data.

## Funding statement

Ibrahim M. Hezam was supported by "Researchers Supporting Project number (RSP2023R389), King Saud University, Riyadh, Saudi Arabia".

## Declaration of interest's statement

The authors declare no conflict of interest.

## Data availability statement

No data was used for the research described in the article.

## Appendix A Preliminaries related to SVNNs

Definition 1 [22]. A SVNS *G* on a finite universal set Ω is mathematically presented as

$$G=\left\{\left\langle x,tm_{G}\left(x\right),im_{G}\left(x\right),fm_{G}\left(x\right)\right\rangle :x\in \Omega \right\}\text{,}$$

wherein $tm_{G}\left(x\right),im_{G}\left(x\right),fm_{G}\left(x\right):\Omega \;\to \;\left[0,1\right]$ with $0\leq tm_{G}\left(x\right)+im_{G}\left(x\right)+fm_{G}\left(x\right)\leq 3,\;\forall \;x\in \Omega \text{.}$ The triplet $\left(tm_{G}\left(x\right),im_{G}\left(x\right),fm_{G}\left(x\right)\right)$ is called “single-valued neutrosophic number (SVNN)” and signified by $\alpha =\left(tm_{\alpha },im_{\alpha },fm_{\alpha }\right)$.

Definition 2 [22]. Let $\alpha_{1}=\left(tm_{\alpha_{1}},\;im_{\alpha_{1}},\;fm_{\alpha_{1}}\right),\;\alpha_{2}=\left(tm_{\alpha_{2}},\;im_{\alpha_{2}},\;fm_{\alpha_{2}}\right)\in SVNNs\left(\Omega \right)$ and λ>0. Then the operational laws on SVNNs are given as

• $\alpha_{1}^{c}=\left(fm_{\alpha_{1}},1-im_{\alpha_{1}},tm_{\alpha_{1}}\right)$,
• $\alpha_{1}\subseteq \alpha_{2}$ if $tm_{\alpha_{1}}\leq tm_{\alpha_{2}},im_{\alpha_{1}}\geq im_{\alpha_{2}}$ and $fm_{\alpha_{1}}\geq fm_{\alpha_{2}}$,
• $\alpha_{1}=\alpha_{2}$ if and only if $\alpha_{1}\subseteq \alpha_{2}$ and $\alpha_{1}⊇\alpha_{2}$,
• $\alpha_{1}+\alpha_{2}=\left(tm_{\alpha_{1}}+tm_{\alpha_{2}}-tm_{\alpha_{1}}.\;tm_{\alpha_{2}},\;im_{\alpha_{1}}.im_{\alpha_{2}},\;fm_{\alpha_{1}}.fm_{\alpha_{2}}\right)$,
• $\alpha_{1}\;.\alpha_{2}=\left(tm_{\alpha_{1}}.tm_{\alpha_{2}},\;im_{\alpha_{1}}+im_{\alpha_{2}}-im_{\alpha_{1}}.\;im_{\alpha_{2}},\;fm_{\alpha_{1}}+fm_{\alpha_{2}}-fm_{\alpha_{1}}.\;fm_{\alpha_{2}}\right)$,
• $\lambda \;\alpha_{1}=\left(1-{\left(1-tm_{\alpha_{1}}\right)}^{\lambda },\;im_{\alpha_{1}}^{\lambda },\;fm_{\alpha_{1}}^{\lambda }\right)$,
• $\lambda \;\alpha_{1}=\left(tm_{\alpha_{1}}^{\lambda },1-{\left(1-im_{\alpha_{1}}\right)}^{\lambda },1-{\left(1-fm_{\alpha_{1}}\right)}^{\lambda }\right)$.

Definition 3 [27]. For a SVNN $\alpha =\left(tm_{\alpha },im_{\alpha },fm_{\alpha }\right)\text{,}$ the score function is presented by

(A1) $$S\left(\alpha \right)=\frac{2+tm_{\alpha }-im_{\alpha }-fm_{\alpha }}{3},\;S\left(\alpha \right)\in \left[0,1\right]\text{.}$$

Definition 4 [83]. Consider $\alpha_{j}=\left(tm_{\alpha_{j}},im_{\alpha_{j}},fm_{\alpha_{j}}\right)$ be a set of SVNSs and $\iota ={\left(\iota_{1},\iota_{2},...,\iota_{n}\right)}^{T}$ be the weights' set of $\alpha_{j}$ satisfying $\iota_{j}\in \left[0,1\right]$ and $\sum_{j=1}^{n}\iota_{j}=1\text{.}$ Then the “single-valued neutrosophic weighted average (SVNWA)” and “single-valued neutrosophic weighted geometric (SVNWG)” operators are presented as

(A2) $$SVNWA\left(\alpha_{1},\alpha_{2},...,\alpha_{n}\right)=\overset{n}{\underset{j=1}{⊕}}\;\iota_{j}\;\alpha_{j}=\left(1-\prod_{j=1}^{n}{\left(1-tm_{j}\right)}^{\iota_{j}},\prod_{j=1}^{n}{\left(im_{j}\right)}^{\iota_{j}},\prod_{j=1}^{n}{\left(fm_{j}\right)}^{\iota_{j}}\right),$$

(A3) $$SVNWG\left(\alpha_{1},\alpha_{2},...,\alpha_{n}\right)=\overset{n}{\underset{j=1}{⊗}}\;\alpha_{j}^{\iota_{j}}=\left(\prod_{j=1}^{n}{\left(tm_{j}\right)}^{\iota_{j}},1-\prod_{j=1}^{n}{\left(1-im_{j}\right)}^{\iota_{j}},1-\prod_{j=1}^{n}{\left(1-fm_{j}\right)}^{\iota_{j}}\right)\text{.}$$

Definition 5 [84]. Let $\alpha_{1},\alpha_{2}\in SVNNs\left(\Omega \right)\text{.}$ Then the distance measures for SVNSs is given by

(A4) $$D_{h}\left(\alpha_{1},\alpha_{2}\right)=\frac{1}{3m}\;\sum_{i=1}^{m}\left(\left|tm_{\alpha }\left(x_{i}\right)-tm_{\alpha_{2}}\left(x_{i}\right)\right|+\left|im_{\alpha_{1}}\left(x_{i}\right)-im_{\alpha_{2}}\left(x_{i}\right)\right|+\left|fm_{\alpha_{1}}\left(x_{i}\right)-fm_{\alpha_{2}}\left(x_{i}\right)\right|\right)\text{.}$$

Definition 6 [85]. Suppose θ:(0,1]→R is continuous and strictly decreasing. Then a strictly Archimedean t-norm is given by

$$\delta \left(x,x^{'}\right)=\theta^{-1}\left(\theta \left(x\right)+\theta \left(x^{'}\right)\right),\;for\;x,\;x^{'}\in (0,1]\text{.}$$

Definition 7 [85]. Suppose ψ:(0,1]→R is a continuous mapping satisfying ψ(l)=θ(1−l),l∈(0,1] and ψ is strictly increasing. Then a strictly Archimedean *t*-conorm is defined by

$$\rho \left(x,x^{'}\right)=\psi^{-1}\left(\psi \left(x\right)+\psi \left(x^{'}\right)\right),\;for\;x,\;x^{'}\in (0,1],$$

The *t*-norm and *t*-conorm operations, initiated by Dombi (1982) are usually called the Dombi operations and are given by

Definition 8 [86]. For $x,\;y\in \left[0,1\right],$ the Dombi *t*-norm and *t*-conorm can be described by

DOM(x,y)=11+{(1−xx)k+(1−yy)k}1k,

DOMcx,y=11+x1−xk+y1−yk1k,k≥1.

These operations have well-mannered precedence of variation over parameter *‘k’*.
