# Supplementary material for: An extended DNMA-based multi-criteria decision-making method and its application in the assessment of sustainable location for a lithium-ion batteries’ manufacturing plant
Source: Heliyon. 2023 Mar 7;9(3):e14244. doi: 10.1016/j.heliyon.2023.e14244 (PMC10010990; doi:10.1016/j.heliyon.2023.e14244)
Supplement: Multimedia component 1 [file mmc1.docx]

**Supplementary File**

**S.1 Archimedean-Dombi operations on SVNNs**

**Definition 1:** Consider the SVNNs Assume that and where and Then we discuss the ADOs on SVNNs given as

(i)

(S1)

(ii)

(S2)

(iii)

(S3)

(iv)

(S4)

Some special cases (for *k* = 1) of the presented operations are depicted as follows:

**Case-I:** For where we have

(i)

(ii)

(iii)

(iv)

which are elementary algebraic operations on SVNNs*.*

**Case-II:** For where we have

(i)

(ii)

(iii)

(iv)

which are Einstein operations on SVNNs*.*

**Case-III:** For where we have

(i)

(ii)

(iii)

(iv)

which are elementary Hamacher operations on SVNNs*.*

**Theorem 1:** Consider the SVNNs and . Then we have

(i)

(ii)

(iii)

(iv)

(v)

(vi)

**Proof:** (i)-(ii) Proofs are obvious.

(iii) In accordance with Definition 9, we obtain

Next, we have

Thus,.

**S.2 SVN-ADWA Operators**

**Definition 2:** Consider be a group of SVNSs and be the weights of such that and Then, we define the SVN-ADWA operator as

(S5)

**Theorem 2:** The aggregation of is also a SVNN. Moreover, we have

(S6)

**Proof:** The 1^st^ part follows from Definition 2. Next, to establish the 2^nd^ part, the method of mathematical induction on *n* is used, which is demonstrated below.

For *n*=1, we have

Thus Eq. (S6) is satisfied when *n*=1. Suppose Eq. (S6) is satisfied for *n*=*R*. Then,

Now for *n*=*R+*1, we have

Thus, Eq. (S6) is satisfied for *n*=*R*+1 also. Thus, Eq. (S6) is true for all natural numbers *n*.

A few special cases of the *SVNADWA* operator are given by

**Case-I:** If where then the *SVN-ADWA* operator reduces to the “SVN-weighted averaging (*SVNWA*)” operator

(S7)

**Case-II:** If where then the *SVN-ADWA* operator transforms to the “SVN-Einstein weighted averaging (SVNEWA)” operator is shown as

(S8)

**Case-III:** If where then Eq. (S6) reduces to the “SVN-Hamacher weighted averaging (SVNHWA) operator” as

(S9)

Next, we extract the following results:

**Property 1:** (*Shift invariance*) If is a SVNN, then

**Property 2:** (*Idempotency*) If is a SVNN, then .

**Property 3:** (*Boundedness*) Let be a set of SVNNs. Then, we have where .

**Property 4:** (*Monotonocity*) If be two sets of SVNNs satisfying then

**Definition 3:** Consider $\alpha_{j} = \left(tm_{j}, im_{j}, fm_{j}\right)$ be a set of SVNSs and be the weights of with the condition and Then we define the “single-valued neutrosophic-Archimedean-Dombi geometric (SVN-ADWG)” operator as

(S10)

**Theorem 3:** The aggregation of is also a SVNN. Moreover, we have

(S11)

Some particular cases of the SVN-ADWG operator are given by

**Case-I:** If where then (A15) reduces to the “SVN-weighted geometric (SVNWG) operator” as

(S12)

**Case-II:** If where then (S11) converts to the “SVN-Einstein weighted geometric (SVNEWG) operator” as

(S13)

**Case-III:** If where then (S11) reduces to the “SVN-Hamacher weighted geometric (SVNHWG) operator” as shown below:

(S14)

Further, we extract the following results:

**Property 5:** (*Shift invariance*) If is a SVNN, then

**Property 6:** (*Idempotency*) If is a SVNN, then .

**Property 7:** (*Boundedness*) Let be the set of SVNNs. Then, we have where

.

**Property 8:** (*Monotonocity*) If being two collections of SVNNs satisfying , then
